# Spatial Organization of the Sperm Cell Glycoproteome

**DOI:** 10.1016/j.mcpro.2024.100893

**Published:** 2024-12-12

**Authors:** Rensong Ji, Riccardo Zenezini Chiozzi, Henk van den Toorn, Miguel Leung, Tzviya Zeev-Ben-Mordehai, Nathan D. Burke, Elizabeth G. Bromfield, Karli R. Reiding, Albert J.R. Heck

**Affiliations:** 1 Biomolecular Mass Spectrometry and Proteomics, Bijvoet Center for Biomolecular Research and Utrecht Institute for Pharmaceutical Sciences, Utrecht University, Utrecht, The Netherlands; 2 Netherlands Proteomic Center, Utrecht, The Netherlands; 3 Division of Biosciences, Institute of Structural and Molecular Biology, University College London, London, United Kingdom; 4 Division of Biosciences, University College London Mass Spectrometry Science Technology Platform, University College London, London, United Kingdom; 5 Structural Biochemistry, Bijvoet Center for Biomolecular Research, Utrecht University, Utrecht, The Netherlands; 6 School of BioSciences, Faculty of Science, Bio21 Institute, University of Melbourne, Parkville, VIC, Australia; 7 Infertility and Reproduction Research Program, School of Environment and Life Sciences, The University of Newcastle, Callaghan, NSW, Australia; 8 Department of Biomolecular Health Sciences, Utrecht University, Utrecht, The Netherlands

## Abstract

Sperm cells are terminally differentiated cells that are essential for reproduction in sexually reproducing species. Consistent with their highly specialized function, sperm cells harbor a unique proteome containing many proteins not expressed in somatic cells. In contrast, the post-translational landscape of the sperm proteome remains largely unexplored, limiting our understanding of how modifications such as glycosylation impact sperm function and sperm-egg interactions. Here, we used glycopeptide-centric glycoproteomics to comprehensively characterize protein *N*-glycosylation in sperm from three mammalian species, revealing clear conservation of glycosylation profiles. We find that glycosylation patterns in sperm proteins are distinct from those in plasma, with as clear distinctive features less sialyation and more paucimannosylation in sperm. Moreover, based on their subcellular location, sperm protein glycosylation varies, with paucimannose species enriched in the acrosomal vesicle, oligomannose species in the sperm head membrane, and complex glycan species in the acrosomal membrane.

## Highlights

• The glycoproteome of human, bull, and boar sperm are analyzed.
• The sperm glycoproteome is very distinct from the plasma glycoproteome.
• Sperm cells are highly compartmentalized and so are its glycan characteristics.
• Paucimannose glycans are enriched in the acrosomal vesicle, oligomannose glycans in the head membrane, and complex glycans in the acrosomal membrane.
• Glycoproteins and glycan characteristics, as well as their compartmentalization, are conserved between the studied organisms.

## Graphical Abstract

**Figure:**
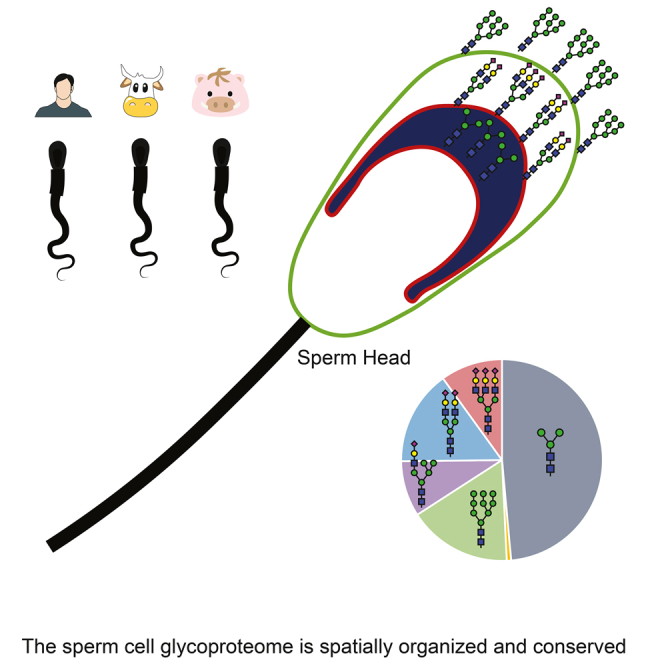

## In Brief

Here, we analyzed protein glycosylation in sperm cells from human, bull, and boar without using any glycopeptide enrichment as this may introduce a bias. Sperm cells are highly compartmentalized and possess a specialized acrosome, a lysosome-like organelle. We demonstrate that also the sperm cell glycoproteome is spatially organized and highly conserved across the three studied species, with paucimannose glycans enriched in the acrosomal vesicle, oligomannose glycans in the head membrane, and complex glycans in the acrosomal membrane.

Mature sperm are highly polarized cells whose streamlined architecture is specialized for finding and fusing with the egg. Sperm cells have a head and a tail, which can be further subdivided into sub-compartments (1). The sperm head consists mainly of the nucleus and an organelle called the acrosome, a lysosome-like vesicle whose exocytosis in the female reproductive tract remodels the sperm surface and renders it capable of fusing with the egg plasma membrane (2, 3, 4). The tail consists of a microtubule-based axoneme surrounded by several sperm-specific accessory structures, the extents of which define the sub-compartments of the tail. In the most proximal part of the tail, known as the midpiece, mitochondria wrap into a spiral sheath around the axoneme. In the principal piece, the axoneme is surrounded by a unique cytoskeletal structure called the fibrous sheath, which is thought to both physically reinforce the sperm tail and to support metabolism by anchoring glycolytic enzymes (5).

The composition and subcellular distribution of the sperm proteome has been studied extensively in a range of species (6, 7, 8, 9, 10, 11, 12). However, significantly less is known about the post-translational landscape of sperm proteins (13, 14, 15), although glycosylation has been implicated in sperm survival within the female reproductive tract (16) and in sperm-egg interactions leading to fusion (17). Indeed, glycans play key roles in regulating protein-protein interactions across a wide range of host-pathogen and cell-cell recognition events (18, 19), and aberrant glycosylation is linked to human disease (20). Thus, proper glycosylation is likely to be crucial for sperm function, but the lack of comprehensive studies on the sperm glycoproteome limit our understanding of how sperm glycans contribute to male (in)fertility. For instance, although the human sperm glycoproteome has been previously characterized (15), we do not know how specific glycan features are distributed across subcellular compartments within the sperm cell, or whether these features are conserved across species.

Here, we comprehensively analyzed the *N*-glycoproteomes of human, bull, and boar sperm cells and compare them to the glycoproteome of human plasma. Not surprisingly, we find that the glycan characteristics of human sperm cells are distinct from those of human plasma, with as distinctive features less sialyation and more paucimannosylation in sperm. More importantly, we find that the distinct sperm sub-compartments possess distinct glycan signatures, and that these patterns are conserved across the species studied here.

## Experimental Procedures

### Chemicals, Reagents, and Samples

Tris-HCl, Triton X-100, tris(2-carboxyethyl)phosphine (TCEP), chloroacetamide (CAA), MgCl_2_, sodium deoxycholate, Dulbecco’s phosphate-buffered saline (DPBS), sodium orthovanadate, and trypsin (product number T1426) were purchased from Sigma-Aldrich. DNase I was purchased from ThermoFisher Scientific. Benzonase, phosphoSTOP phosphatases inhibitors, and cOmplete Mini EDTA-free protease inhibitors were purchased from Merck Millipore. Acetonitrile was acquired from Biosolve. Lys-C protease was purchased from Roche Diagnostics Nederland BV. Urea was purchased from VWR international B.V. Percoll was obtained from GE Healthcare.

Commercial human plasma (Visucon-F frozen normal control plasma) was purchased from Affinity Biologicals. Boar (*Sus scrofa*) sperm samples were obtained from Varkens KI Nederland ('s-Hertogenbosch, The Netherlands). Frozen bull (*Bos taurus*) semen straws were obtained from the Utrecht University Veterinary Faculty. Three male (*Homo sapiens*) semen samples were obtained from heathy normozoospermic donors acquired with written consent provided in accordance with the University of Newcastle's Human Ethics Committee guidelines (Approval No. H-2013-0319) abiding by the Declaration of Helsinki principles. Sperm samples from all species (human, bull, and boar) were prepared as previously reported (21, 22, 23). Pooled samples were used to be representative for each species, thereby limiting inter-individual variation. In short, high quality human sperm cells were acquired using Percoll density gradients and fractionated by centrifugation at 500*g*, then pooled for use, while bull and boar sperm cells were centrifuged through a discontinuous gradient of 35% and 70% Percoll at 750*g*. From each sample, approximately 50 × 10^6^ cells were brought into a new tube and spun down at 13, 800*g* for 10 min at 4 °C, after which the supernatant was removed.

### Cell Lysis and Digestion

To limit a potential bias due to sample preparation, all samples were prepared at the same time using the same protocol and buffers following the protocol from Leung, *et al.* (21). Briefly, 500 μl of cold lysis buffer (100 mM Tris-HCl pH 8.5, 7 M Urea, 1% Triton X-100, 5 mM TCEP, 30 mM CAA, 10 U/ml DNase I, 1 mM MgCl_2_, and 1% benzonase, 1 mM sodium orthovanadate, phosphoSTOP phosphatases inhibitors, and cOmplete Mini EDTA-free protease inhibitors) was added to each sample. Samples were sonicated on ice for 2 min using an ultrasonic processor (UP100H, Hielscher) at 80% amplitude. The proteins were then quantified with a BCA assay and 100 μg of each sample was precipitated with methanol/chloroform. The dried protein pellet was resuspended in digestion buffer (100 mM Tris-HCl pH 8.5, 1% sodium deoxycholate, 5 mM TCEP, and 30 mM CAA). Trypsin and Lys-C proteases were mixed and added to a 1:25 and 1:100 ratio (*w/w*), respectively, and protein digestion was performed overnight at 37 °C. The final peptide mixtures were desalted with solid-phase extraction C18 columns (Sep-Pak, Waters).

### Liquid Chromatography with Tandem Mass Spectrometry (LC-MS/MS)

Approximately 2000 ng of peptides from each sample was injected using an UltiMate 3000 high-performance liquid chromatography system (Thermo Fisher Scientific) coupled online to an Orbitrap Fusion Lumos (Thermo Fisher Scientific). Each sample was analyzed with 3 different MS methods while the chromatography was kept constant.

For the chromatography, buffer A consisted of water with 0.1% formic acid, while buffer B was 80% acetonitrile and 20% water with 0.1% formic acid. The peptides were first trapped for 1 min at 30 μl/min with 100% buffer A on a trap column (0.3 mm by 5 mm with PepMap C18, 5 μm, 100 Å; Thermo Fisher Scientific); after trapping, the peptides were separated by a 50-cm analytical column packed with C18 beads (Poroshell 120 EC-C18, 2.7 μm; Agilent Technologies). The gradient was 9% to 45% B in 100 min at 300 nl/min. Buffer B was then raised to 55% in 5 min and increased to 99% for the cleaning step.

For each MS method, peptides were ionized using a spray voltage of 2 kV and a capillary heated at 275 °C. The mass spectrometer was set to acquire full-scan MS spectra (from *m*/*z* 350–1400 Th) for a maximum injection time of 120 ms at a mass resolution of 120,000 and an automated gain control (AGC) target value of 1 × 10^6^. The dynamic exclusion was set to 45 s for an exclusion window of 10 ppm with a cycle time of 2 s. Charge-states screening was enabled, and precursors with 2+ to 8+ charge states and intensities >5 × 10^4^ were selected for MS^2^. All samples were initially fragmented in the HCD cell, with the readout in the Orbitrap mass analyzer at a resolution of 15,000 (isolation window of 2 Th) and an AGC target value of 1 × 10^5^ with the maximum injection time set to 22 ms and a normalized collision energy (NCE) of 30%. One of the three methods only had this initial fragmentation, while the other two methods had an extra step meant to maximize glycoproteomics readout. On detection of at least 3 ions from a list of oxonium ions (Supplemental Table S1) (24), either stepping-collision energy HCD (sHCD) MS^2^ with 20%, 28%, and 36% NCE or electron-transfer higher-energy collisional dissociation (EThcD) MS^2^ was triggered (isolation window of 2 Th) and fragment ions (*m/z* 120–4000) were analyzed in the Orbitrap mass analyzer at a resolution of 30,000 (AUTO injection time), with AGC target value of 1 × 10^5^. With slight adjustment on the resolution of the mass analyzer, or charge state screening, or NCE of each method, each sample were analyzed nine times in total using these three MS methods.

### Data Analysis

All raw files obtained with all methods were first analyzed with MaxQuant version 2.1 with all default settings except for adding deamidation (N) as dynamic modification and activating the match-between-runs option. Trypsin/P was defined as protease and a maximum of 2 missed cleavages were allowed for the database search. The main search peptide tolerance was set to 4.5 ppm and the peptide fragment mass tolerance to 20 ppm. Carbamidomethylation (C) was set as a fixed modification, while deamidation (N), acetyl (protein N-term), and oxidation (M) were set as dynamic modifications. A false discovery rate of 0.01 was set for peptides and proteins. For each sample, we used the specific protein library file that contained the reference proteomes downloaded from UniProt (25), all within February 2022. The human database contained 79,057 entries, the bull database 37,519 entries, and the boar database 49,793 entries. With this initial search, we were able to calculate intensity-based absolute quantification (iBAQ) values and created 3 smaller FASTA files with less than 3300 entries to use for glycan-specific analysis. Homolog (glyco)proteins of human, bull, and boar were determined based on protein sequence in the database by a two-way blast algorism and considered matching with E-values lower than 1 × 10^−4^ (Supplemental Table S2) (26). Unless specified otherwise, homolog proteins of the species have the same name.

For glycoproteomics analysis, we used Byonic (version v5.0.3, Protein Metrics Inc) to identify glycopeptides. The precursor mass tolerance was set to 10 ppm and the fragment mass tolerance to 20 ppm. The databases used were the protein databases from the proteomics search and a customized glycan database. The protein databases contained 461, 3224, 2095, and 2531 proteins for human plasma, human, bull, and boar sperm cells, respectively. The customized glycan database was initially made based on the monosaccharide building blocks indicated by oxonium ions in MS/MS spectra (Supplemental Table S1) and the current understanding of the biosynthesis of *N*-glycans in mammals (27), which includes 4600 glycan composition (Supplemental Table S3). Then, using this expansive glycan database, pilot searches were conducted for one file of each of the species, after which the results were inspected manually. Only glycan characteristics with evident MS/MS annotations and at least 5 PSMs were retained (Supplemental Table S4), resulting in a curated glycan database of 892 glycan composition comprising HexNAc, Hex, phosphorylation, dHex, NeuAc, acetylation, and formylation (Supplemental Table S5). NeuGc-containing glycan compositions were not included in the curated glycan database to minimize the search time, based on the finding that there was only one PSM supporting its potential existence in bull sperm cells in the (time-consuming) pilot search. Glycans in this database consisted of a combination of deoxyhexose (fucose), hexose (glucose, mannose, galactose), *N*-acetylhexosamine (*N*-acetylglucosamine), *N*-acetylneuraminic acid, and phosphate, as well as their derivatives with acetylation and/or formylation. A single glycan composition was allowed per glycopeptide as common modification, and cysteine (Cys) carbamidomethylation was set as a fixed modification. Next to this, single phosphorylation of serine (Ser), threonine (Thr), or tyrosine (Tyr) residues, pyroglutamate formation of protein N-terminal glutamine (Gln) and glutamic acid (Glu), acetylation of the protein N-terminus, and up to two events of oxidation of methionine (Met) and/or tryptophan (Trp) were allowed as variable modifications. Cleavage sites were set to arginine (Arg) and lysine (Lys). The enzyme activity was set to semi-specific, with at most 1 miscleavage to ensure manageable search time. Search results were curated for having a |log probability| ≤1.5, a score threshold of ≥150, and a delta mod score ≥10 (28).

Peptide-spectrum matches (PSMs) were used to quantify the glycopeptides (Supplemental Table S6). Peptide-spectrum matches of individual glycan composition were counted using an in-house script. Glycan composition with formylation was added to their non-formylated version of glycan composition because formylation was an artifact caused by using formic acid during sample preparation (29). Glycan composition was classified into six categories of characteristics: unoccupied (no monosaccharides on a given site), paucimannose (HexNAc <3 and Hex <4), phosphomannose (Phospho >0), oligomannose (HexNAc = 2 and Hex >3), hybrid/asymmetric (HexNAc = 3), di-antennary (HexNAc = 4), and extended (HexNAc >4) (30).

Glycoproteins were clustered using hierarchical clustering based on their glycan characteristics. Glycan characteristics of proteins were the sum of glycan characteristics of their glycopeptide-spectrum matches (glycoPSMs). Then, the classified glycoPSMs were normalized using min-max normalization and were further used for clustering (Supplemental Table S5). Euclidean distance was used to determine the distance of each data point, and Ward’s method was used to measure the distance between clusters. The resulting groups of glycoproteins were subjected to gene ontology (GO 2023-07-27) analysis to find out whether they localized in a certain subcellular localization (31). Four was decided as the number of clustered groups because GO analysis annotated all four groups for boar and three out of four groups for human and bull. When multiple subcellular locations were annotated for a protein cluster, the subcellular localization with the lowest *p* value was chosen as representative.

### Data Visualization

We followed the recommendations of the Consortium for Functional Glycomics to depict glycan images using Glycoworkbench (32, 33). Data analysis was performed in Rstudio using the following packages: ‘openxlsx’, ‘dplyr’, ‘maditr’, ‘tidyr’, ‘caret’, ‘factoextra’, ‘stringr’, ‘ape’, ggdendro’, ‘ggplot2’, and ‘readxl’.

### Experimental Design and Statistical Rationale

Each biological sample underwent analysis nine times with three different mass spectrometry methods while maintaining consistent chromatography conditions. This resulted in a total of 36 LC-MS raw data files (4 samples × 9 runs) for peptide identification through MaxQuant and glycopeptide identification *via* Byonic. *p*-values were calculated based on the nine technical replicates, with a significance threshold set at less than 0.05. Glycoproteins with similar glycan compositions were grouped into four clusters using hierarchical clustering. Each glycoprotein group was then analyzed for gene ontology cellular components. Protein homology assessments were conducted based on sequence similarity, with E-values set below 1 × 10^−4^.

## Results

We performed *N*-glycoproteomics on mature, ejaculated sperm cells from three mammalian species (human, bull, and boar). For reference, for method optimization, and for a general comparison we also analyzed in parallel the *N*-glycoproteome of pooled human plasma. Cells were lyzed, and released proteins were reduced, alkylated, and digested by LysC and trypsin, followed by desalting. The resulting tryptic peptides were analyzed by LC-MS/MS with HCD fragmentation, as well as with oxonium-ion triggered sHCD, and EThcD methods. To establish sperm cell proteomes, only the HCD data were used and searched against the reference proteomes of each species (UniProt database, downloaded in February 2022) using MaxQuant.

In-depth, glycoproteomics studies are typically performed following specific enrichment of glycopeptides by, *e.g.*, hydrophilic-interaction liquid chromatography (HILIC) solid-phase extraction (SPE). However, we and others have shown that this form of enrichment may lead to a bias introduced by the loss of glycopeptides that harbor small glycans, i.e., paucimannose and even smaller, and thereby introduce a systemic bias in the observed glycoproteome (18, 19), as documented for neutrophil azurophilic granules, a lysosome-like compartment. As sperm cells contain acrosomes that are also thought to be lysosome-like we made the choice to perform N-glycoproteomics without prior enrichment (18).

### Sperm Cell Proteomes Overlap Between Species but are Distinct From Plasma

For human plasma, human, bull, and boar sperm cells, respectively, 4191, 23,802, 14,245, and 17,775 peptides were identified, which could be matched to 461, 3224, 2095, and 2531 proteins (Table 1). The structural components of the sperm tail, namely, the A-kinase anchor protein 4 (AKAP4) and the outer dense fiber protein 1 (ODF1), were quantified to be two of the most abundant proteins shared between human, bull and boar sperm cell proteomes (Supplemental Table S2), in line with previous observations (14, 34). In human plasma, albumin, hemopexin, immunoglobulins (immunoglobulin heavy constant gamma 1 and 2), and apolipoproteins were the most abundant proteins, again in accordance with the literature (35).

**Table 1:** Overview of (glyco)peptides and (glyco)proteins identification

Identification	Human plasma	Human sperm	Bull sperm	Boar sperm
Identified peptides	4191	23,802	14,245	17,775
Identified proteins	461	3224	2095	2531
Identified glycopeptides	8229	5429	4935	8102
Identified glycoproteins	66	73	61	84

**Fig. 1:**
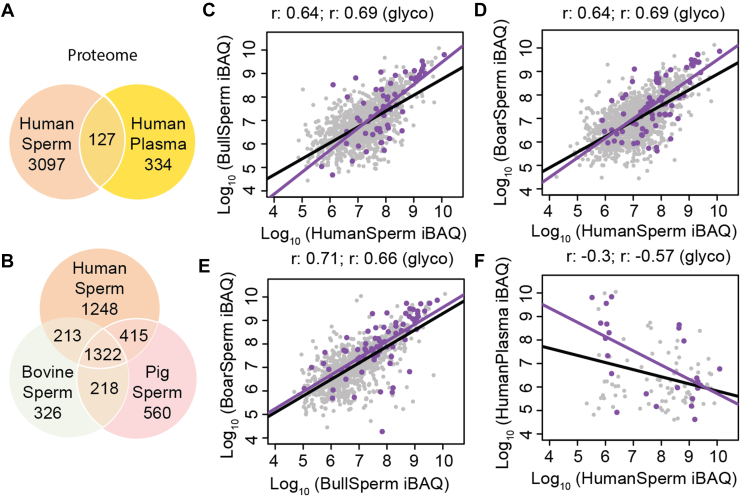
**Proteome and glycoproteome of sperm cells (human, bull, and boar).***A*, number of, and overlap in, detected proteins between human plasma and human sperm, (*B*) number of, and overlap in, detected proteins between human, bovine and boar sperm, (*C*) correlation in protein abundance between human and boar sperm cells, (*D*) correlation in protein abundance between human and boar sperm cells, (*E*) correlation in protein abundance between bull and boar sperm cells, (*F*) correlation in protein abundance between human plasma and human sperm cells. r: Pearson correlation coefficient. All abundances were estimated from log_10_(iBAQ) values from label-free proteomics data. *Gray dots*: proteome; *purple dots*: glycoproteome.

Unsurprisingly, only a small part (4%) of the human sperm cell proteome was shared with human plasma (Fig. 1*A*). In contrast, many of the human sperm cell proteins (∼41%) had clear orthologs in both bull and boar sperm cells (Fig. 1*B*). Another proportion of the sperm cell proteins were only identified in one specific species (39% for human, 16% for bull, and 22% for boar), with another group of proteins only shared between two species (7% between human and bull, 13% between bull and boar, and 10% between boar and human) (Fig. 1*B*). The partial overlap and non-overlap observed here may be due to genuine unique species-dependent proteins, but also be caused by the threshold set for calling two proteins homologs (E-value <1 × 10^−4^) (26). Additionally, it could be attributed to our proteomics analysis potentially capturing only a portion of the complete sperm proteome and expansion/change of UniProt proteome database, resulting in certain homologs genuinely present in sperm cells not being detected in our study, such as tubulin alpha-3D chain, L-lactate dehydrogenase A-like 6A, and histone H3.X. For instance, the study published in 2022 identified 5685 sperm proteins among which 3667 were not identified in our study (14).

### Glycan Characteristics of Sperm cell N-Glycoproteomes are Conserved Between Species

To investigate the sperm cell *N*-glycoproteomes, we searched the sperm cell and plasma sample set using Byonic with a customized *N*-glycan database. The glycan database was generated by (1) checking MS/MS data to determine monosaccharide building blocks (2), iterating across biologically reasonable *N*-glycan composition based on the current understanding of *N*-glycobiosynthesis (27), and (3) a pilot search with manual check to prepare a non-redundant *N*-glycan database with acceptable search times. In the end, we retained 892 potential *N*-glycan compositions (Supplemental Table S4) ranging from paucimannose to complex tetra-antennary species, allowing additionally modifiers such as fucosylation and acetylation. All glycoproteomics LC-MS/MS data, including the results including three fragmentation methods, were used to search for *N*-glycopeptides. Only glycoproteins with at least 5 glycoPSMs were retained for further analysis.

In total, 7229, 5429, 4935, and 8102 glycopeptides were identified in human plasma, human, bull, and boar sperm cells, respectively, which could be matched to 66, 73, 61, and 84 glycoproteins (Table 1). The most abundant human, bull, and boar sperm cell glycoproteins were AKAP4, sperm acrosome membrane-associated protein 1 (SACA1), and zona pellucida-binding protein 1 (ZPBP1). The correlation coefficient of the log(iBAQ) values between all proteins/glycoproteins detected in sperm cells were 0.64/0.69, 0.64/0.69, and 0.71/0.66 (Fig. 1, *C–E*), indicating a relative high correlation of glycoprotein abundances in the sperm cells of the three species. As expected, and as negative control, no correlation was observed in glycoprotein abundances between human plasma and sperm cells (Fig. 1*F* and Supplemental Table S7).

**Fig. 2:**
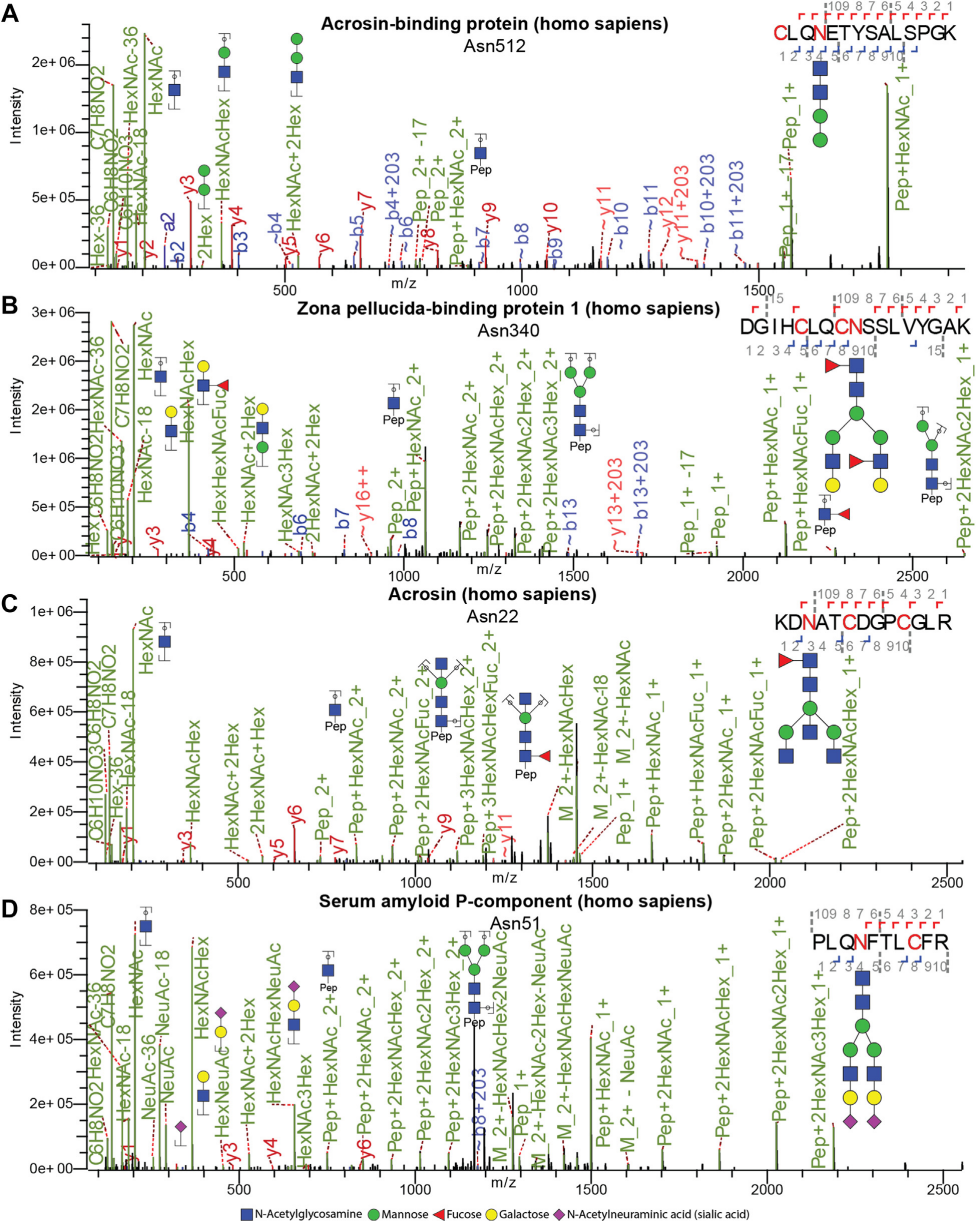
**Annotated MS/MS spectra of representative characteristic glycopeptides.** In each depicted MS/MS spectra a glycopeptide from human sperm cell was chosen that carried specific type of glycans, namely (*A*) paucimannosylation, (*B*) core- and antennary-fucosylation, (*C*) bisection, and (*D*) sialylation. Monosaccharides are represented as: *N*-acetylhexosamine (GlcNAc; *blue square*), mannose (Man; *green circle*), fucose (Fuc; *red triangle*), galactose (Gal; *yellow circle*), and *N*-acetylneuraminic acid (NeuAc; dark magenta diamond). In the peptide sequence, the glycosylated asparagine and the alkylated cysteine are highlighted in *red*. Since mass spectrometry primarily does not primarily differentiate between glycan isomers, the suggested glycan structures are based on literature estimates. Glycan cartoons of the fragment ions are positioned pointing upwards while those of the parent composition are positioned pointing downwards.

Our MS/MS data supported a plethora of different glycosylation features, namely, paucimannosylation (Fig. 2*A* and Supplemental Fig. S1), core- and antennary-fucosylation (Fig. 2*B* and Supplemental Fig. S2), LacdiNAc antenna (Supplemental Fig. S3), bisection (Fig. 2*C* and Supplemental Fig. S4, *A* and *B*), sialylation (Fig. 2*D*, Supplemental Figs. S3*B* and S4*C*), and phosphomannosylation (Supplemental Fig. S5). Except for paucimannosylation, which did not have a unique oxonium ion (B-ion) and was solely confirmed by glycopeptide mass, these glycan features could all be confirmed by the annotation of the corresponding signature fragment ions of HexHexNAcdHex and DGIHCLQCNSSLVYGAK-HexNAcdHex (*m/z* 512.20 [M+H]^+^and *m/z* 2271.04 [M+H]^+^, respectively), NeuAc (*m/z* 292.10 [M+H]^+^), KDNATCDGPCGLR-3HexNAcHex (*m/z* 1118.47 [M+3H]^3+^), phosphoHex (*m/z* 243.03 [M+H]^+^), and 2HexNAc (*m/z* 407.17 [M+H]^+^), respectively. The *N*-glycan structures were drawn by assembling all the annotated B-ions and Y-ions following the rules of eukaryotic *N*-glycobiosynthesis. It was noted that the types of *N*-glycans mentioned above were found in the sperm cells of all species. For comparison, in human plasma we only found evident annotations for oligomannose species, LacNAc antenna, sialylation, bisecting GlcNAc, core- and antennary-fucosylation (Supplemental Figs. S6 and S7), which was in line with previous studies (36, 37), hinting at distinct and broader glycosylation features in sperm cells.

**Fig. 3:**
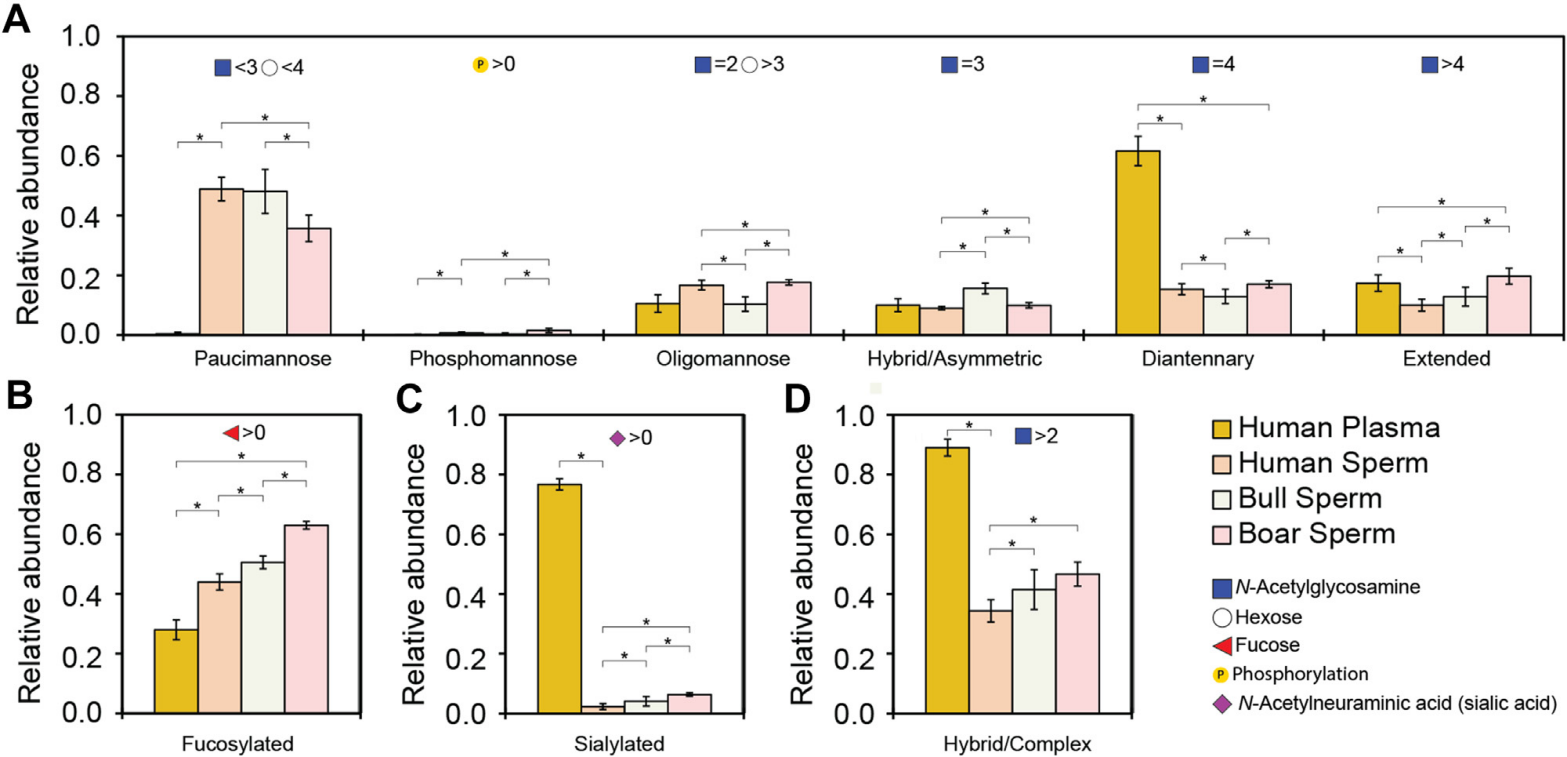
**Overview of *N*-Glycan characteristics observed in human- bull- and boar-sperm cells and for comparison in human plasma.** Glycan characteristics were categorized as, (*A*) paucimannose, phosphomannose oligomannose, hybrid/asymmetric, diantennary, extended, (*B*) fucosylated, (*C*) sialylated, and (*D*) hybrid/complex. The relative abundance of these glycan features was identified using Byonic. The glycan characteristics were combined for each injection and normalized, then, the mean and standard deviation of the number were calculated using nine independent injections, and a two-tail homoscedastic *t* test were performed between samples. The bars show the mean ± standard deviation of the technical replicates of nine injections with different MS methods. ∗ indicates a *p*-value < 0.05.

Combining the individual glycan composition into general characteristics such as fucosylation level and antennarity, we could compare the sperm cell samples and human plasma in a more comprehensive way. Overall, while the glycan characteristics of human, bull, and boar sperm cells were different in the statistical sense (*p* < 0.05), since the largest delta difference across all glycan characteristics (14%) was three times smaller than that between human plasma and sperm (48%), we interpreted this as high similarity in glycosylation features among the sperm cell samples (Fig. 3). For glycan characteristics of sperm cells, an average of 40% were paucimannose, less than 1% were phosphomannose, 14% were oligomannose, 12% were hybrid, 15% were diantennary, and 12% were extended. A much larger difference could be seen between human sperm cells and human plasma, the latter predominantly having diantennary (62%) sialylated (77%) glycosylation and less than 1% paucimannose species. Moreover, it was noted that 44% of human sperm cell glycans were fucosylated, ∼1.6 times more than that in human plasma (28%) (*p* < 0.001). Even while more than 34% of human sperm cell glycans carried complex antennae, less than 3% of these were found to be sialylated, a 26-fold difference compared to human plasma (77%) (*p* < 0.001). The most abundant glycan composition observed in human plasma, human, bull, and boar sperm cells were HexNAc_4_Hex_5_NeuAc_2_, HexNAc_2_Hex_3_, HexNAc_2_Hex_3_dHex_1_, and HexNAc_2_Hex_3_dHex_1_, respectively. A full comparison of the observed glycans can be found in Supplemental Fig. S8 and Supplemental Table S8. Multi-fucosylation (fucose >2), indicative of antennary fucosylation, was previously reported to be an abundant feature in sperm cells (15, 36). In our study, multi-fucosylated glycans were detected, but only in a small amount (less than 4%). To illustrate this further, the glycoPSMs of glycopeptides carrying two fucoses (150 for human sperm cells) was much smaller than the glycoPSMs of glycopeptides carrying one fucose (2242 for human sperm cells).

### Protein N-Glycan Characteristics are Defined by Their Subcellular Location

**Fig. 4:**
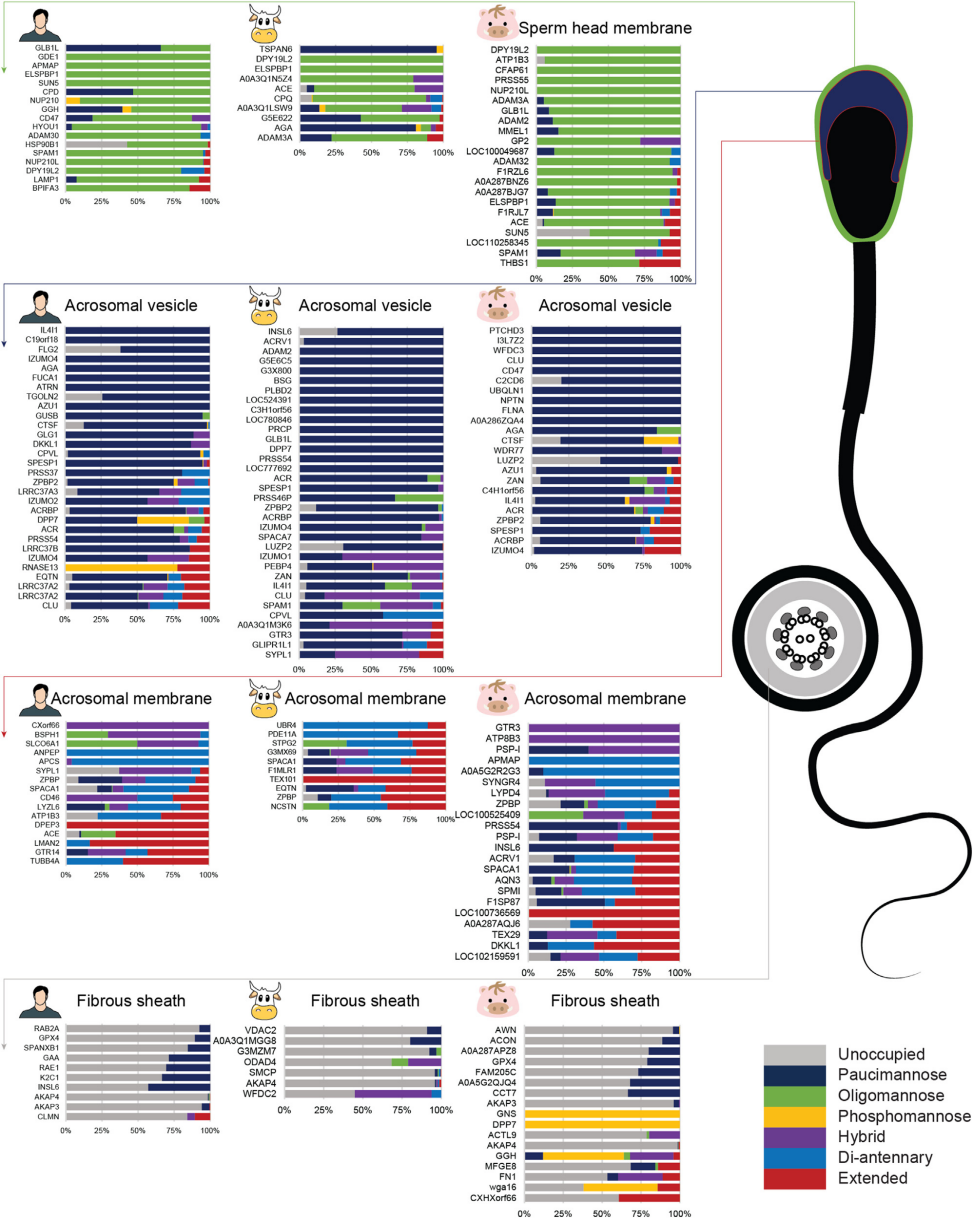
**Human (*left*), bull (*middle*), and boar (*right*) sperm cell glycoproteins with alike *N*-glycan features display alike subcellular localizations.** Glycan characteristics were categorized as being paucimannose (HexNAc <3 and Hex <4), phosphomannose (Phospho >0), oligomannose (HexNAc = 2 and Hex >3), hybrid/asymmetric (HexNAc = 3), di-antennary (HexNAc = 4), and extended (HexNAc >4). Whereas sperm head contains glycoproteins that are primarily harboring oligomannose glycans (*green*), the acrosomal vesicles feature glycoproteins carrying primarily paucimannose glycans (*dark blue*). Di-antennary (*blue*) and extended (*red*) glycans decorate mostly glycoproteins being part of the acrosomal membrane. Glycoproteins were arranged based on the size of the glycan using the equation: unoccupied × 0+paucimannose × 10^0^+phosphomannose × 10^1^+oligomannose × 10^2^+hybrid × 10^3^+diantennary × 10^4^+extended × 10^5^. Subcellular localizations were chosen base on lowest *p*-value, sperm head membrane (*p*-value = 5 × 10^–10^ for boar sperm), acrosomal vesicle (*p*-values = 1 × 10^–13^, 2 × 10^–16^, 2 × 10^–5^ for human, bull, and boar sperm cells, respectively), acrosomal membrane (*p*-values = 2 × 10^–6^, 6 × 10^–5^, 7 × 10^–6^), and fibrous sheath (*p*-values = 5 × 10^–7^, 3 × 10^–5^ (midpiece), 2 × 10^–5^). Principally, proteins are listed by their gene name. For those proteins without gene name, UniProt entry name or identifiers were used instead.

Through unsupervised hierarchical clustering, we clustered human, bull, and boar sperm cell glycoproteins into four groups based on their glycan characteristics (Fig. 4 and Supplemental Table S9). These characteristics are 1) paucimannose, 2) oligomannose, 3) unoccupied, and 4) hybrid/complex glycans. Besides representative glycans, other types of glycans were present in all groups as well, for instance, in the group with dominant paucimannose glycans, glycoproteins like clusterin (CLUS), equatorin (EQTN), and dipeptidyl peptidase 2 (DPP7) also had unoccupied sites, oligomannose species, and di-antennary species. However, these features were present in lower amounts so that the main glycan characteristics could be attributed to the respective groups with high confidence. These four groups of glycoproteins were subsequently analyzed by GO cellular component analysis. To confirm whether this spatial organization of the sperm cell glycosylation is conserved among human, bull, and boar, the subcellular location of all the 22 homolog glycoproteins of human, bull, and boar were manually cross-referenced to literature covering their experimentally-determined localizations (Supplemental Table S10). Interestingly, GO-term analysis significantly assigned the four groups to distinct locations (FDR *p* < 0.05, Supplemental Table S9), namely the sperm head plasma membrane (*p* value = 5 × 10^−10^, oligomannose), the acrosomal vesicle (*p* value = 1 × 10^−13^, 2 × 10^−16^, and 2 × 10^−5^, paucimannose), the fibrous sheath for human and boar (*p* value = 5 × 10^−7^ and 7 × 10^−6^, unoccupied) or sperm midpiece for bull (*p* value = 3 × 10^−5^, unoccupied), and the acrosomal membrane (*p* value = 1 × 10^−5^, 5 × 10^−5^, and 5 × 10^−10^, hybrid/complex), respectively (Fig. 4). Human and bull sperm cell glycoproteins with oligomannose species were not unambiguously assigned to a particular subcellular location by GO-term analysis (DFR *p* > 0.05) because these glycoproteins are from various cell membranes such as nuclear membrane (Probable C-mannosyltransferase DPY19L2) (8), endoplasmic reticulum lumen (endoplasmin) (38), lysosome membrane (lysosome-associated membrane glycoprotein 1) (39). Therefore, they were less significantly assigned to sperm plasma membrane for human sperm (*p* value = 6 × 10^−5^, FDR = 6 × 10^−2^) and membrane (*p* value = 5 × 10^−1^, FDR = 1). As these human and bull glycoproteins were mostly homologs to boar glycoproteins assigned to the sperm head membrane, we also assumed that these human and bull sperm cell glycoproteins with oligomannose species are localized in the sperm head membrane.

It must be noted that the four groups of glycan characteristics were not exclusively present in only one specific subcellular location, since GO-term analysis typically gave more than one location with *p* values lower than 1 × 10^−4^ (see Supplemental Table 8 for the full overview). This is not surprising as for instance, the angiotensin-converting enzyme (ACE) can be in the sperm head membrane (where ACE helps sperm epididymal transit and sperm-zona pellucida binding), and in the midpiece of the tail (where ACE contributes to sperm cell motility) (40). Here, the subcellular location with the most confident *p* value was chosen. As expected, homolog glycoproteins were found to be localized at the same subcellular location (for example, epididymal sperm-binding protein 1, ELSPBP1, localized in sperm head membrane, CLUS localized in acrosomal vesicle, and ZPBP localized in acrosomal membrane). However, for some of these this was less clear. For example, hyaluronidase PH-20, SPAM1, was annotated by GO analysis to be in human and boar sperm cell localized in the sperm head membrane while in bull sperm cell SPAM1 seemed to be primarily localized in the acrosomal vesicle.

Altogether, our results indicate that the subcellular distribution of glycan profiles in sperm is conserved, at least in mammals. For the sperm cells of all studied species, glycoproteins with dominant paucimannose structures localized in the acrosomal vesicle, glycoproteins with unoccupied sites were predominantly located in the fibrous sheath, while hybrid, di-antennary, and complex species were distributed across various cell membranes. In contrast, glycoproteins with oligomannose composition, localized primarily in the sperm head.

### Protein Glycosylation at the Residue-Specific Level

**Fig. 5:**
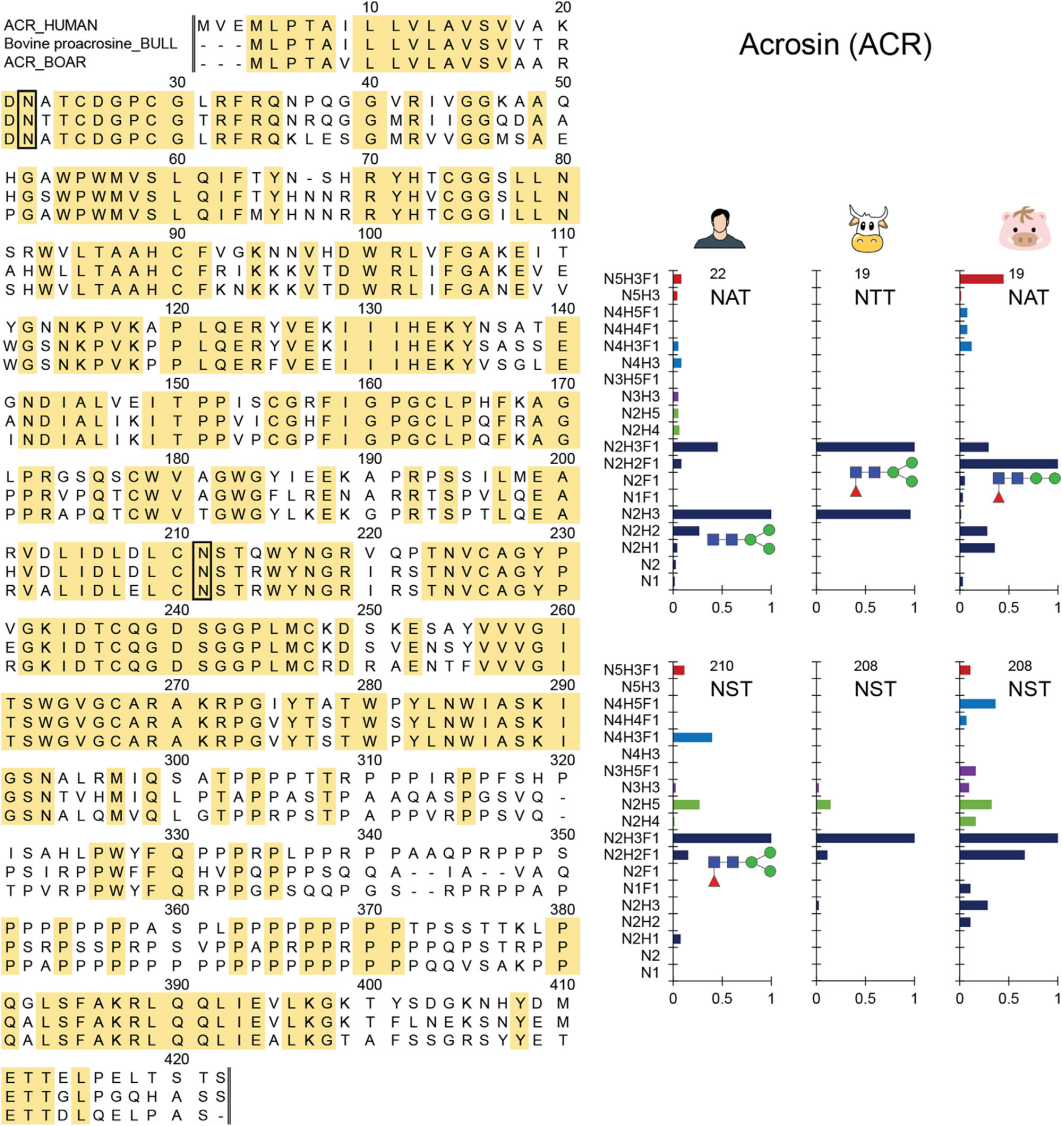
**Sequence alignment and site-specific *N*-glycan profile of the sperm cell glycoprotein acrosin (ACRO).** Acrosin is highly abundant in sperm of human, bull, and boar origin. Acrosin is 69% identical across the three species (calculated by averaging the percent identity matrix) and two of the *N*-glycosylation sites (Asn22 and Asn210 in human) are also conserved. Moreover, the *N*-glycans decorating these two conserved sites are also similar, based on the glycoproteomics data generated here. In this analysis, glycans were only included when at least five glycopeptide PSMs were detected at a glycosylation site. The protein sequences were aligned in Uniprot with zero iterations and the same amino acids at the same sites of human, bull, and boar sperm cell proteins are highlighted in *yellow*. Observed *N*-glycosites are annotated in *black boxes*. Principally, proteins are listed by their gene name. For those proteins without gene name, UniProt entry name or identifiers were used instead.

Among species, zona pellucida binding protein (ZPBP1, Supplemental Fig. S9), SACA1 (Supplemental Fig. S10), acrosin (ACRO, Fig. 5), EQTN (Supplemental Fig. S11), and the nuclear pore membrane glycoprotein 210-like (P210L, Supplemental Figs. S12–S14) were the glycoproteins harboring the most glycoPSMs. These glycoproteins were consequentially selected for closer inspection at the level of individual glycosylation sites (Supplemental Tables S11 and S12). In addition to this, CLUS (Supplemental Fig. S15) was noted to be the sole glycoprotein that was detectable in sperm cells as well as in human plasma. Therefore, we also investigated the differences in *N*-glycosylation in sperm cell and plasma clusterin.

Generally, the glycan characteristics at each site were found to be rather consistent across species. For site annotation we use the human proteins. For example, the sites carrying oligomannose species could systematically be detected in sperm cells across all species, examples being at Asn433 of P210L and at Asn824 of P210L. The same was true for glycosites carrying paucimannose species (*e.g.*, at Asn210 of ACRO), and those with di-antennary or higher complexity (*e.g.*, at Asn101 of EQTN). Some exceptions were found as well, for instance at Asn824 of P210L, in which extended glycan species were observed in human sperm cells but not in bull and boar.

For CLUS, the glycoprotein we detected both in plasma and sperm cells, our human sperm cell data revealed that the top *N*-glycans were fucosylated with one fucose at four out of five identified glycosites, which sharply contrasted with plasma CLUS, in which fucosylation was largely absent. Additionally, human sperm cell CLUS carried primarily paucimannose species, whereas plasma CLUS primarily features di-antennary or complex glycan species. In summary, several of the more dominant glycosylation features and individual *N*-glycosylation sites are quite conserved in sperm cells of different species, hinting at potential functional roles.

## Discussion

The main aim of this study was to investigate the glycoproteome of sperm cells obtained from human, bull, and boar. We have generated a repository of glycoproteomics data that will serve as an important resource for understanding the role of sperm glycans in normal sperm function. Our datasets will also act as references for assessing how glycan profiles are altered in cases of male infertility. This article presents information on glycan composition, with the glycan structures illustrated based on the literature for better visualization. We do not cover details on glycan isomerism or linkage as reported in some studies (41, 42, 43).

Sperm cell protein *N*-glycans have previously been investigated at different molecular levels. This includes released glycan analysis/glycomics (36), probing deglycosylated glycosites (13), and as we do here by analyzing glycopeptides (30). Without exception, in all these studies the small glycans were likely underestimated. In glycomics, small glycans cannot be released by PNGase F and, in glycoproteomics, each glycopeptide enrichment prefers certain glycans, e.g., HILIC enrichment preferably enriches larger glycans. However, from this study it has become apparent that paucimannose glycosylation is a key feature of sperm cell glycoproteins, particularly those that predominantly reside in the acrosome. Although paucimannosidic proteins are associated with locomotion, development, ageing and the generation of alloantibodies, the contribution of paucimannose to their functions has not been investigated (44). Paucimannose has been observed mostly in lysosomes, and due to the co-existing glycosidases, paucimannose has been generally recognized as a by-product of co-existing glycosidases (30).

To prevent misinterpreting the results, we need to keep in mind the systematic limitations in our analysis. For instance, we may have missed orthologs with low sequence identity but that are structurally or functionally similar. Furthermore, although GO analysis is an accepted method to annotate compartmentalization of proteins, it may have its limitations in defining localization, especially in more poorly characterized cells such as sperm. For instance, in our GO analysis TUBB4A is annotated as “acrosomal membrane” but by using manual annotation should be axonemal; VDAC2 and GPX4 are annotated as “fibrous sheath” even though they are associated with mitochondrial membranes (45, 46); and ODAD4 is annotated as “fibrous sheath” but should be axonemal (47). Notwithstanding these limitations, we believe our conclusions that glycosylation patterns in sperm proteins generally vary based on their subcellular location and that these characteristics are conserved across the mammalian species studied are valid.

In total, we identified 1322 sperm cell proteins conserved across human, bull, and boar, among which 87 were *N*-glycosylated. Our data indicated that the top *N*-glycans of sperm-specific homolog glycoproteins were very similar across species, generally matching in complexity. Considering the post-testicular maturation process in the epididymis which involves modification of surface glycoproteins (48), equivalent glycan-modifying enzyme activities are expected in the epididymis as well. The similarity of the *N*-glycan characteristics/composition across species at the levels of detected glycoproteins, site-specific glycan composition, and subcellular localization, hint at their likely significance in sperm function, such as for sperm survival in and migration through the female reproductive tract, or for sperm-egg interaction and fertilization (49, 50, 51). Although the focus here is on *N*-glycosylation, it has been recently demonstrated that sperm cell proteins can also harbor *O*-glycans, primarily consisting of the core 1 type (52). These findings may lead us to study this also in more detail in the future.

Two notable exceptions in our data are ZPBP1 and CLUS. ZPBP1 displayed diverse glycan characteristics across species, namely human ZPBP1 glycans featured primarily paucimannose glycans, bull ZPBP1 carried mostly extended glycans, whereas boar ZPBP1 harbored mostly di-antennary glycans. Since ZPBP1 interacts with the egg coat during fertilization (9), this different glycosylation of ZPBP1 might relate to species-specific sperm-egg binding mechanisms. CLUS is an interesting example as its glycans showed dominant fucosylation in all studied sperm cells but no fucosylation at all in plasma. The different glycosylation of sperm cell and plasma CLUS is because of their different origins. Plasma CLUS is synthesized in most human cells, but sperm cell CLUS primarily originates from the testis, epididymis, and seminal vesicles and adheres to the surfaces of immature, low motile, or morphologically abnormal sperm cells in testis (53, 54). Moreover, different cell types normally generate different glycosylation. Both sperm cell and plasma CLUS have chaperone activity with damaged proteins but fucosylation could enable sperm cell CLUS to send damaged proteins to the dendritic cells through DC-SIGN (55).

Additionally, our data showed that four groups of glycoproteins with alike glycan characteristics localize in the same subcellular compartments. Paucimannose glycans localized to the acrosomal vesicle, oligomannose glycans to the sperm head, and complex glycans to the inner acrosomal membrane, whereas fibrous sheath *N*-glycosylation sites remained primarily unoccupied. Although the exact contribution of glycans to sperm function is so far unclear, the glycoproteins identified in each subcellular locations correlate with literature (Supplemental Table S1) and these glycoproteins play a pivotal role in sperm function, for instance, acrosin is essential for hamster sperm penetration of the zona pellucida (56).

The acrosome is a specialized sperm organelle generated from the Golgi, which is similar to the lysosomes in somatic cells in terms of pH and enzymes it contains (2, 4). Most of the abundant glycoproteins localized in the acrosome were found to be proteases, glycosidases, and binding proteins such as acrosin (56), tissue alpha-L-fucosidase (57), and izumo sperm-egg fusion protein 2 (58). The glycan features in this compartment were predominantly of the paucimannose type, which might reflect the glycosidases present in this compartment. For instance, for human sperm cells, resident mannosidases alpha-mannosidase 2x (MAN2A2) (49) and mannosyl-oligosaccharide 1,2-alpha-mannosidase IA (MAN1A1) (59) can trim oligomannose glycans into paucimannose glycans, while the beta-hexosaminidase HEXA can reduce the presence of antennary GlcNAcs (60). HEXA has been detected at the transcript level in cat sperm cells, indicating the presence of beta-hexosaminidase and, consequently, paucimannose glycans. Furthermore, HEXA is down-regulated in sperm cells from teratospermic cats, hinting at the potential biological significance of paucimannose glycans (61). Interestingly, glycoproteins with phosphomannose could typically undergo dephosphorylation by acid phosphatases after reaching the lysosome (62). Our detection of phosphomannose in the acrosome vesicle could arise from three scenarios: the phosphomannose is an artifact of analyzing the overall glycan composition of the glycoprotein at its designated location, the phosphomannose was not completely dephosphorylated, or acrosomes are more different from lysosomes than the overlap in protein content would suggest.

Inner acrosomal membrane glycoproteins associate with sperm-oocyte interaction (ZPBP1) and fusion (ACRO) (12, 56, 63). The glycoproteins in the acrosomal membrane are found to be primarily glycosylated with complex glycans. Since the acrosome is generated from the Golgi in which complex glycans are produced, it is not unreasonable to detect also complex glycans in the acrosomal membrane (64). As for why acrosomal membrane glycoproteins *N*-glycans were not influenced by the glycosidases in the acrosomal vesicle, we hypothesize that there might be a higher-order arrangement of the enzymes to ensure unintended glycosidase activities or the glycans of the transmembrane glycoproteins faced toward the sperm cytoplasm. Although there are scarce publications demonstrating how their glycans contribute to their functionality, we could infer the importance of the glycans on the functionality of acrosomal membrane glycoproteins from the study of Lu *et al.* that reported on that an impaired *N*-glycosylation process led to the decrease of acrosomal membrane proteins, and consequently, sperm malfunction (65). Additionally, a knockout of MGAT1, which encodes the GlcNAc transferase that initiates complex *N*-glycan synthesis, leads to blocked spermatogenesis and reduced ERK signaling in germ cells. This further hints at the existence and the crucial role that complex glycans can play in sperm cell development (66).

For the fibrous sheath “glycoproteins” we principally detected non-glycosylated sites, examples being the highly abundant A-kinase anchor protein 4 and 3 (AKAP4 and AKAP3). AKAP4 is localized to the sperm principal piece and relocated to the mid-piece and the post-acrosomal region and is associated with capacitation and acrosome reaction through cAMP/PKA/AKAP4 pathway (67, 68). These AKAP proteins are known to be highly phosphorylated. More than 50% of the glycoPSMs of glycoproteins in the fibrous sheath were unoccupied in mature sperm cells, and when they were occupied, more than 50% of the glycoproteins were occupied with paucimannose species. The inherent flexibility of glycans likely precludes their inclusion in the fibrous sheath, which functions to mechanically support the sperm tail (7).

## Conclusion

Here we used a glycopeptide-centric approach to chart the *N*-glycoproteome of sperm cells from human, bull, and boar, using an unbiased approach optimized first on analyzing human plasma. Our data reveal that *N*-glycoproteins are abundant in sperm and conserved across species. We show that characteristics of the sperm *N*-glycoproteome are different from those of human plasma. We also find that distinct compartments of the sperm cell exhibit characteristic *N*-glycosylation features, from which we conclude that the sperm cell glycoproteome is spatially highly organized and conserved across the species studied here. The datasets we provide here will serve as important resources for future studies aimed at dissecting the roles of specific glycans or glycoproteins on sperm function, sperm maturation, and sperm-egg interaction.

## Data Availability

The mass spectrometry data, and all spectra for identified (glyco)peptides in human plasma, human, bull, and boar sperm cells, have been deposited to the ProteomeXchange Consortium (http://proteomecentral.proteomexchange.org) *via* the PRIDE partner repository with the dataset identifier PXD056661.

## Supplemental data

This article contains supplemental data.

## Conflict of interest

The authors declare that they have no conflicts of interest with the contents of this article.
