# Supplementary material for: Spatial Organization of the Sperm Cell Glycoproteome
Source: Mol Cell Proteomics. 2024 Dec 12;24(1):100893. doi: 10.1016/j.mcpro.2024.100893 (PMC11774830; doi:10.1016/j.mcpro.2024.100893)
Supplement: Supplemental Figs. S1–S15 [file mmc2.pdf]

Supplementary Figures for:

Spatial organization of the sperm cell glycoproteome

Rensong Ji[1, 2], Riccardo Zenezini Chiozzi[1,2,3,4], Henk van den Toorn[1,2], Miguel Leung[5], Tzviya Zeev-Ben-Mordehai[5], Nathan D. Burke[6,7], Elizabeth G. Bromfield[6,7,8], Karli R. Reiding[1, 2,*], Albert J.R. Heck[1, 2,*]

1 Biomolecular Mass Spectrometry and Proteomics, Bijvoet Center for Biomolecular Research and Utrecht Institute for Pharmaceutical Sciences, Utrecht University, Padualaan 8, 3584 CH, Utrecht, The Netherlands.

2 Netherlands Proteomic Center, Padualaan 8, 3584 CH, Utrecht, The Netherlands.

3 Institute of Structural and Molecular Biology, Division of Biosciences, Division of Biosciences, University College London, London WC1E 6BT, UK

4 University College London Mass Spectrometry Science Technology Platform, Division of Biosciences, University College London, London, UK

5 Structural Biochemistry, Bijvoet Center for Biomolecular Research, Utrecht University, 3584 CG Utrecht, The Netherlands.

6 School of BioSciences, Faculty of Science, Bio21 Institute, University of Melbourne, Parkville, 3052, VIC, Australia

7 Infertility and Reproduction Research Program, School of Environment and Life Sciences, The University of Newcastle, 2308, NSW, Australia

8 Department of Biomolecular Health Sciences, Utrecht University, Utrecht, The Netherlands

* Correspondence to a.j.r.heck@uu.nl; k.r.reiding@uu.nl

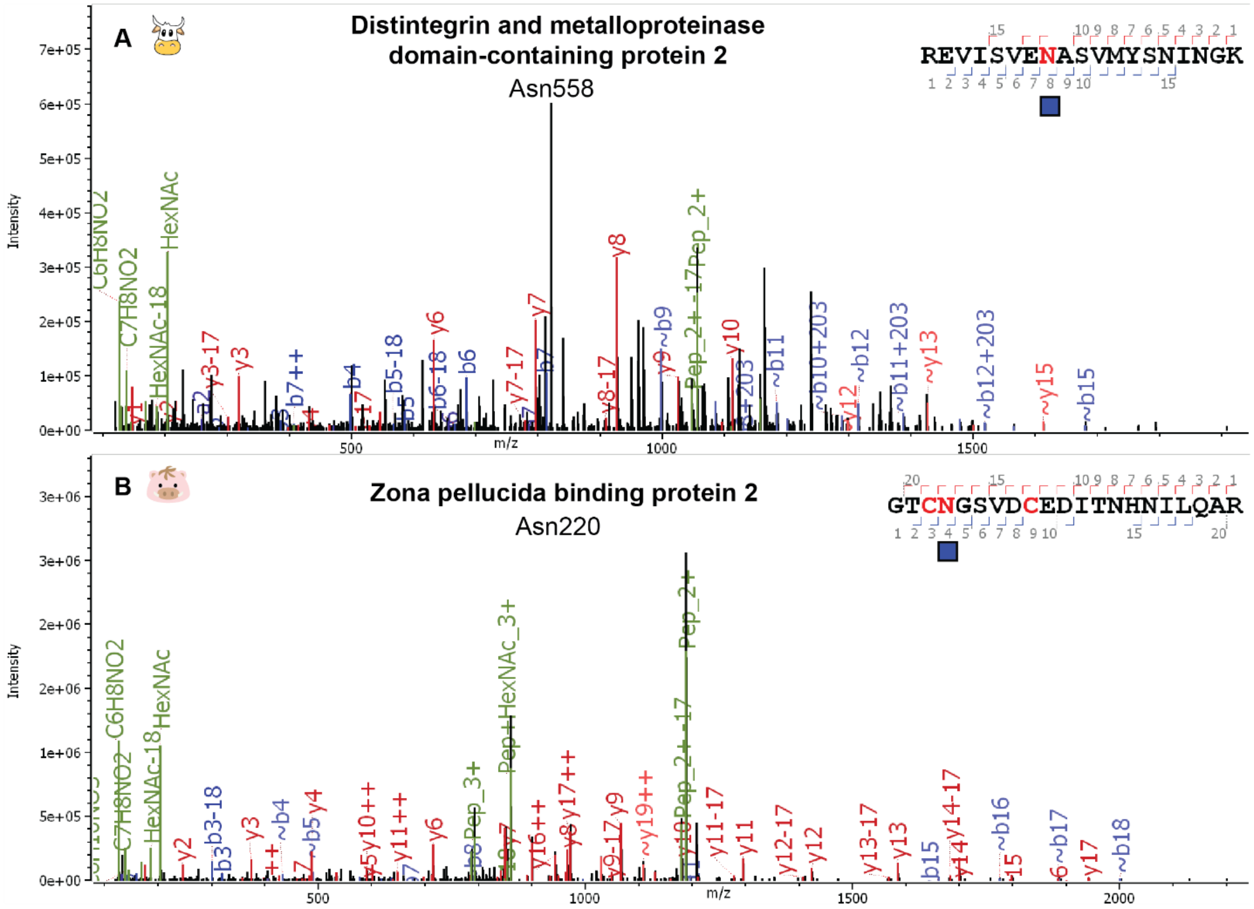

Supplementary Figure S1. Annotated MS/MS spectra of representative glycopeptides carrying only a single HexNAc. In each depicted MS/MS spectra a glycopeptide from bull (A) and boar (B) sperm cell was chosen. Monosaccharides are represented as: *N*-acetylglucosamine (GlcNAc; blue square). In the peptide sequence, glycosylated asparagine and alkylated cysteine are highlighted in red.

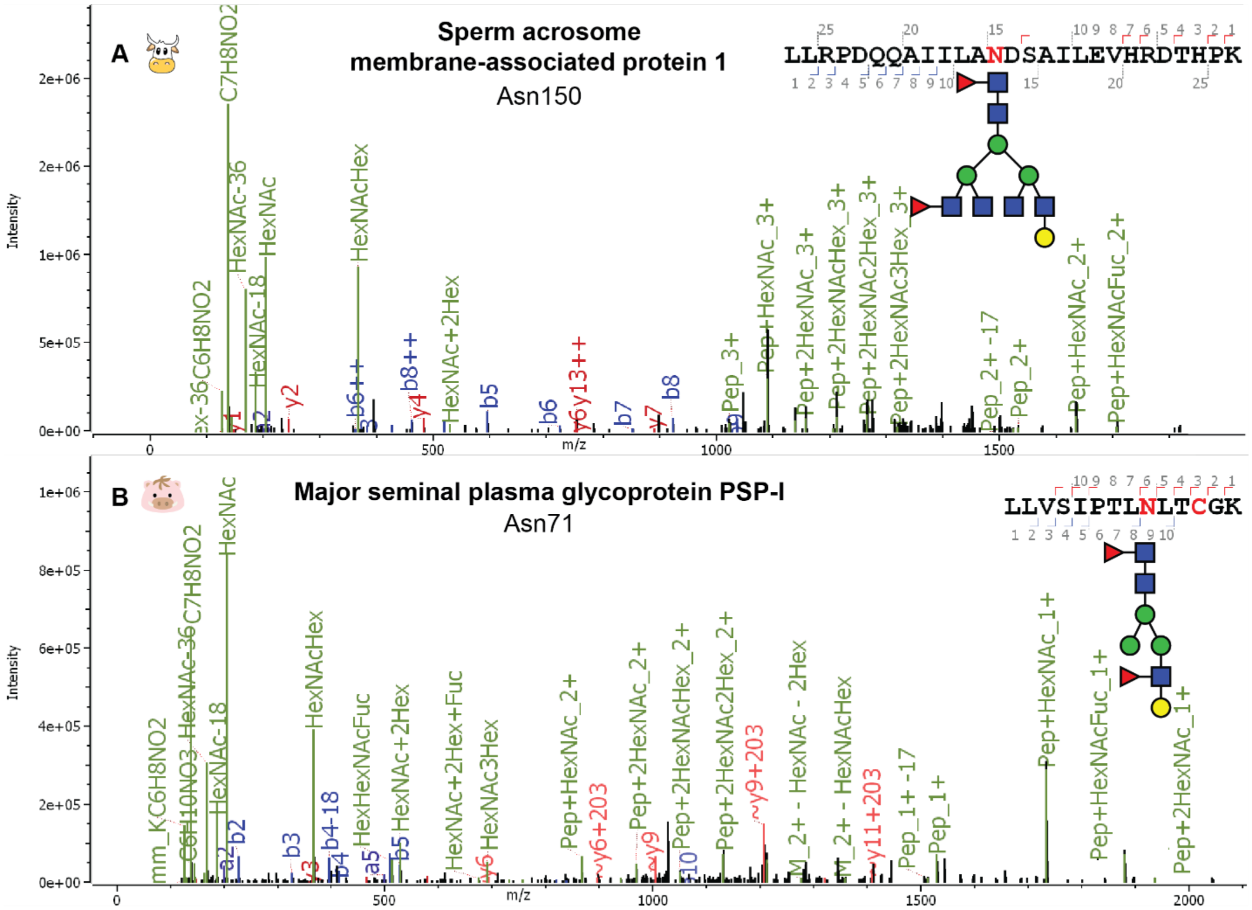

Supplementary Figure S2. Annotated MS/MS spectra of representative glycopeptides carrying core- and antennary-fucosylation. In each depicted MS/MS spectra a glycopeptide from bull (A) and boar (B) sperm cell was chosen that carried core- and antennary-fucosylation. Monosaccharides are represented as: *N*-acetylglucosamine (GlcNAc; blue square), mannose (Man; green circle), fucose (Fuc; red triangle), and galactose (Gal; yellow circle). In the peptide sequence, the glycosylated asparagine and the alkylated cysteine are highlighted in red.

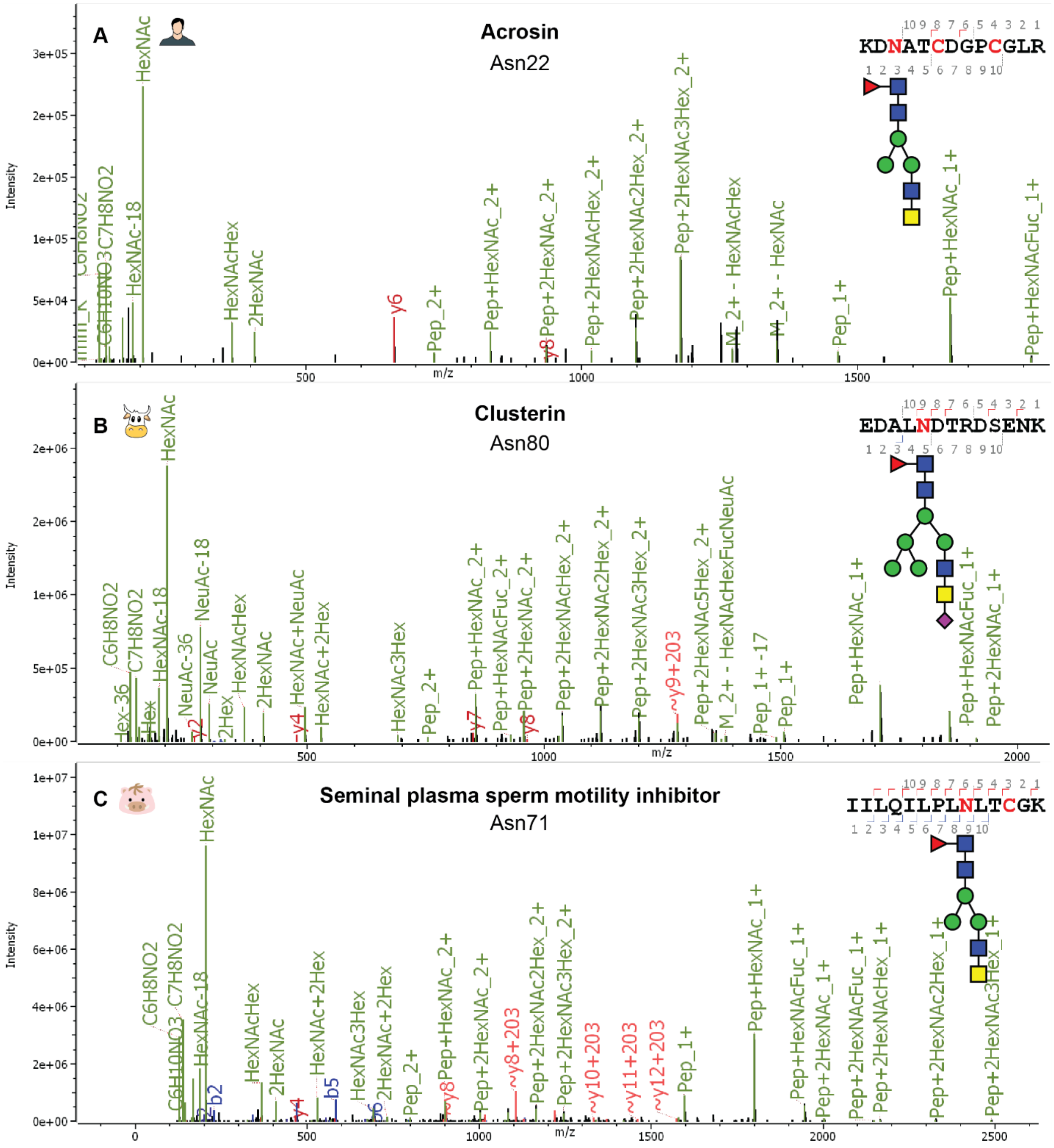

Supplementary Figure S3. Annotated MS/MS spectra of representative glycopeptides carrying LacdiNAc antenna. In each depicted MS/MS spectra a glycopeptide from human (A), bull (B) and boar (C) sperm cell was chosen that carried core-fucosylation and LacdiNAc antenna and/or sialylation. Monosaccharides are represented as: *N*-acetylglucosamine (GlcNAc; blue square), mannose (Man; green circle), fucose (Fuc; red triangle), *N*-acetylgalactosamine (GalNAc; yellow square), and *N*-acetylneuraminic acid (NeuAc; dark magenta diamond). In the peptide sequence, the glycosylated asparagine and the alkylated cysteine are highlighted in red.

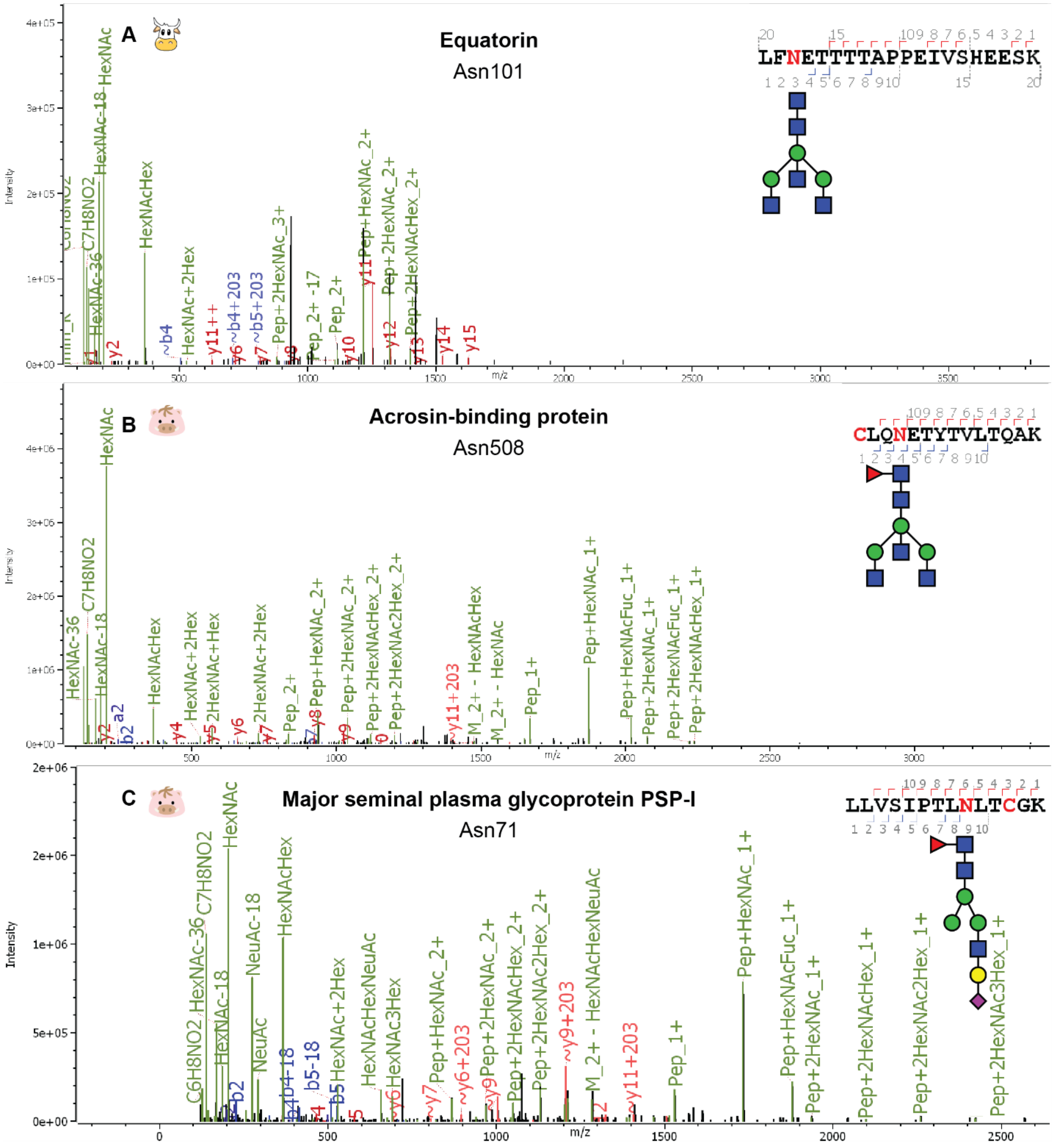

Supplementary Figure S4. Annotated MS/MS spectra of representative glycopeptides carrying bisection or sialylation. In each depicted MS/MS spectra a glycopeptide from bull (A and B) and boar (C) sperm cell was chosen that carried bisection and/or core-fucosylation and/or sialylation. Monosaccharides are represented as: *N*-acetylglucosamine (GlcNAc; blue square), mannose (Man; green circle), fucose (Fuc; red triangle), galactose (Gal; yellow circle), and *N*-acetylneuraminic acid (NeuAc; dark magenta diamond). In the peptide sequence, the glycosylated asparagine and the alkylated cysteine are highlighted in red.

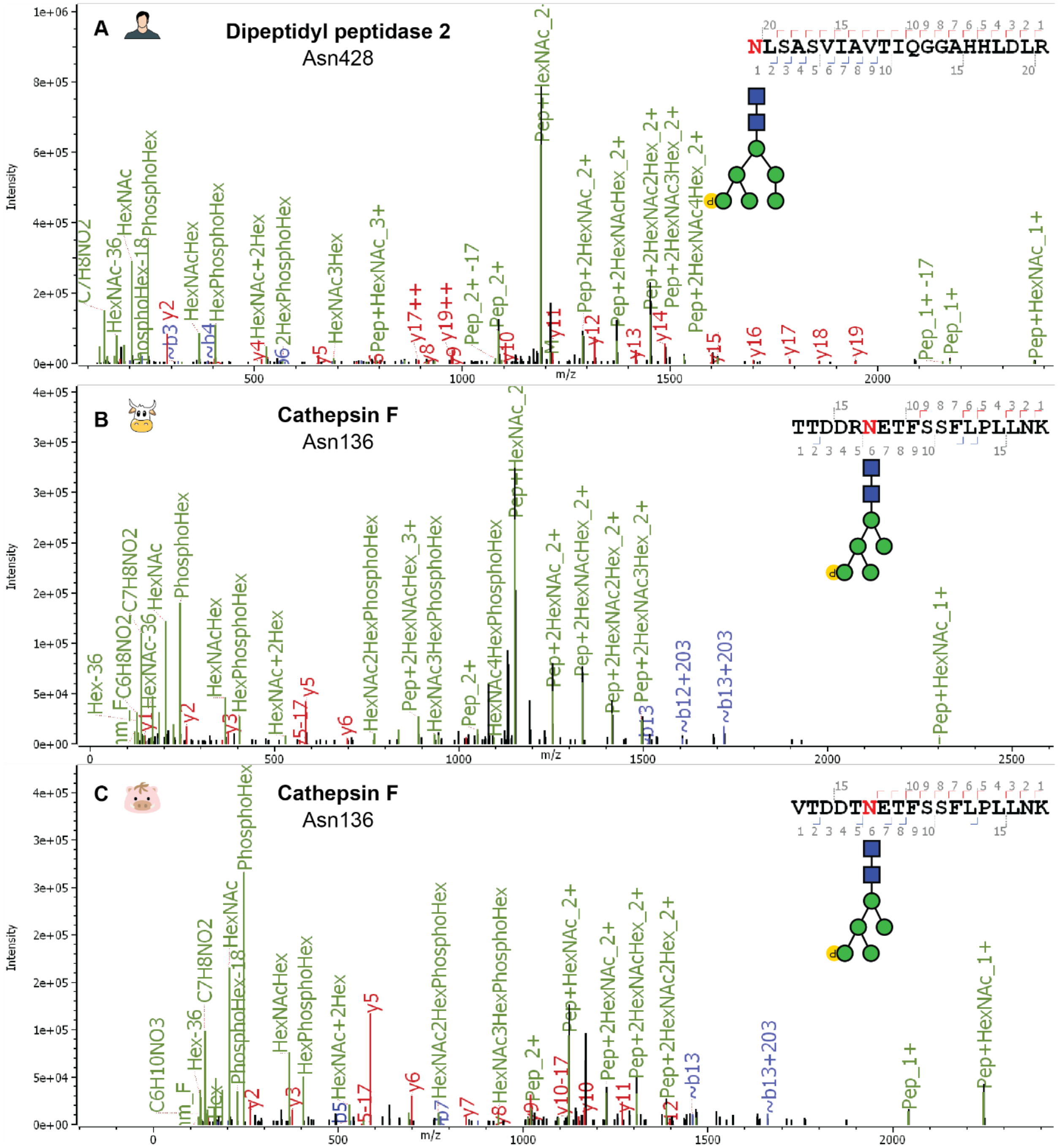

Supplementary Figure S5. Annotated MS/MS spectra of representative glycopeptides carrying oligomannose and phosphomannose. In each depicted MS/MS spectra a glycopeptide from human (A), bull (B), and boar (C) sperm cell was chosen that carried oligomannose and phosphomannose. Monosaccharides are represented as: *N*-acetylglucosamine (GlcNAc; blue square), mannose (Man; green circle), and phosphomannose (phospho; orange circle). In the peptide sequence, the glycosylated asparagine is highlighted in red.

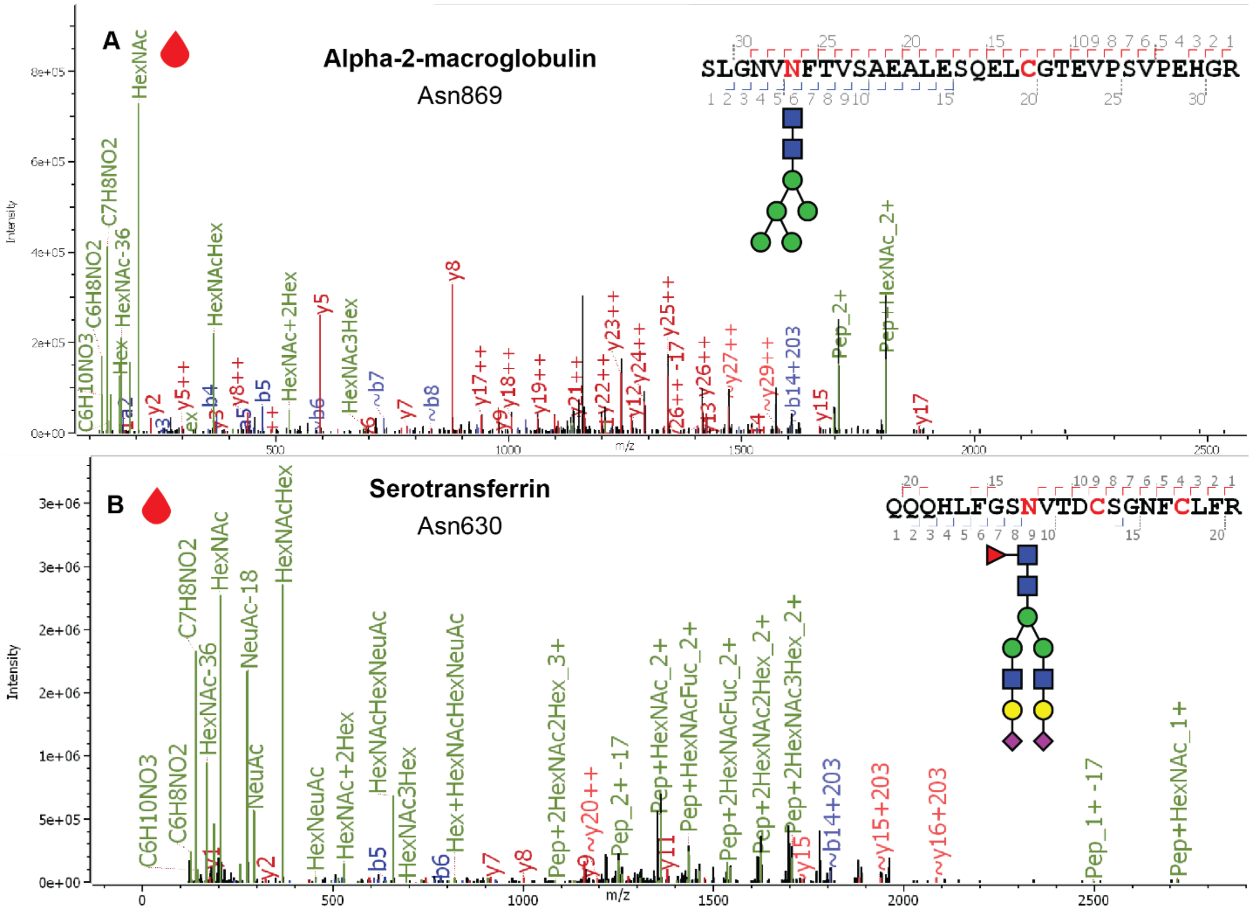

Supplementary Figure S6. Annotated MS/MS spectra of representative glycopeptides carrying oligomannose, core-fucosylation, and sialylation of human plasma. In each depicted MS/MS spectra a glycopeptide from human plasma was chosen that carried (A) oligomannose and (B) sialylation and core-fucosylation. Monosaccharides are represented as: *N*-acetylglucosamine (GlcNAc; blue square), mannose (Man; green circle), fucose (Fuc; red triangle), galactose (Gal; yellow circle), and *N*-acetylneuraminic acid (NeuAc; dark magenta diamond). In the peptide sequence, the glycosylated asparagine and the alkylated cysteine are highlighted in red.

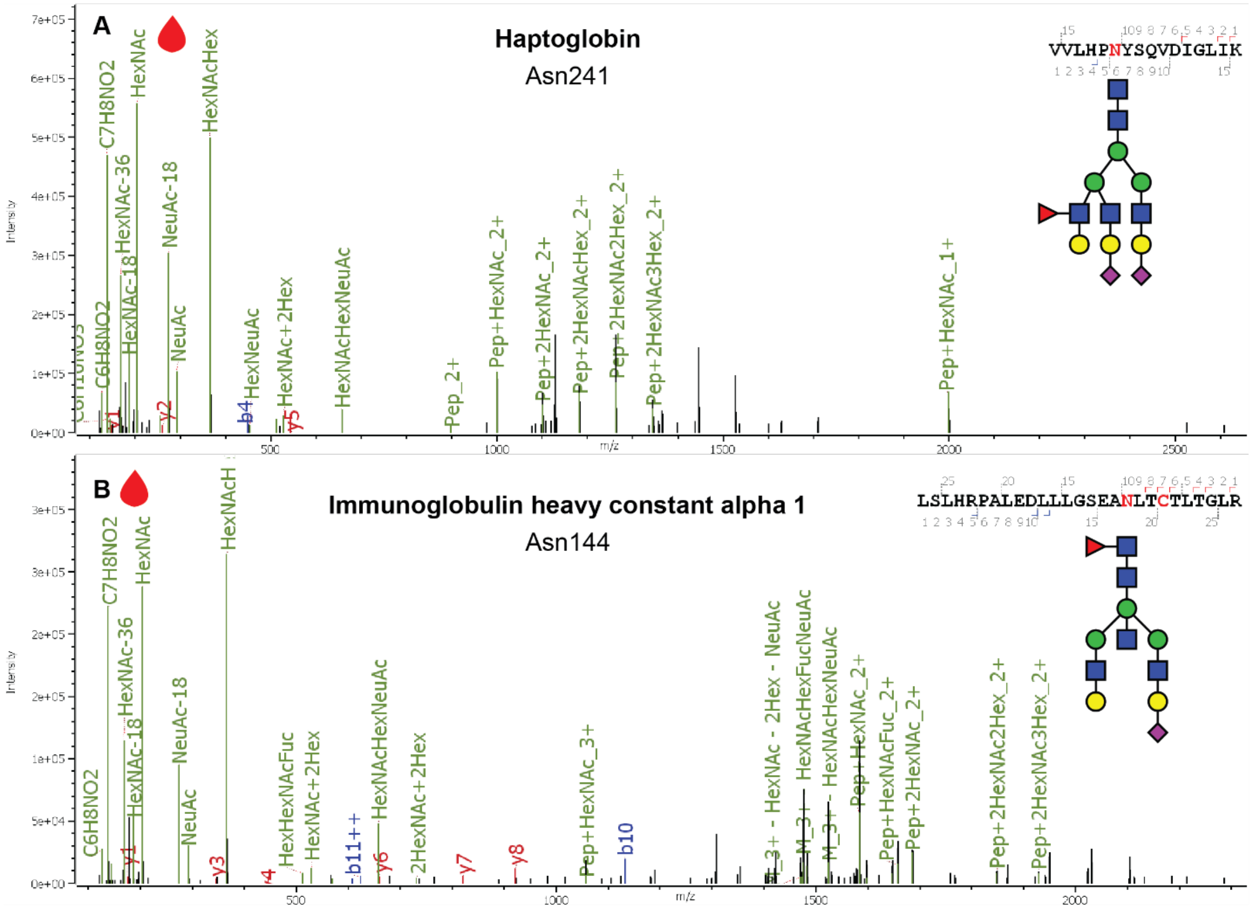

Supplementary Figure S7. Annotated MS/MS spectra of representative glycopeptides carrying antennary-fucosylation and bisection of human plasma. In each depicted MS/MS spectra a glycopeptide from human plasma was chosen that carried (A) antennary-fucosylation and sialylation and (B) core-fucosylation, bisection, and sialylation. Monosaccharides are represented as: *N*-acetylglucosamine (GlcNAc; blue square), mannose (Man; green circle), fucose (Fuc; red triangle), galactose (Gal; yellow circle), and *N*-acetylneuraminic acid (NeuAc; dark magenta diamond). In the peptide sequence, the glycosylated asparagine and the alkylated cysteine are highlighted in red.

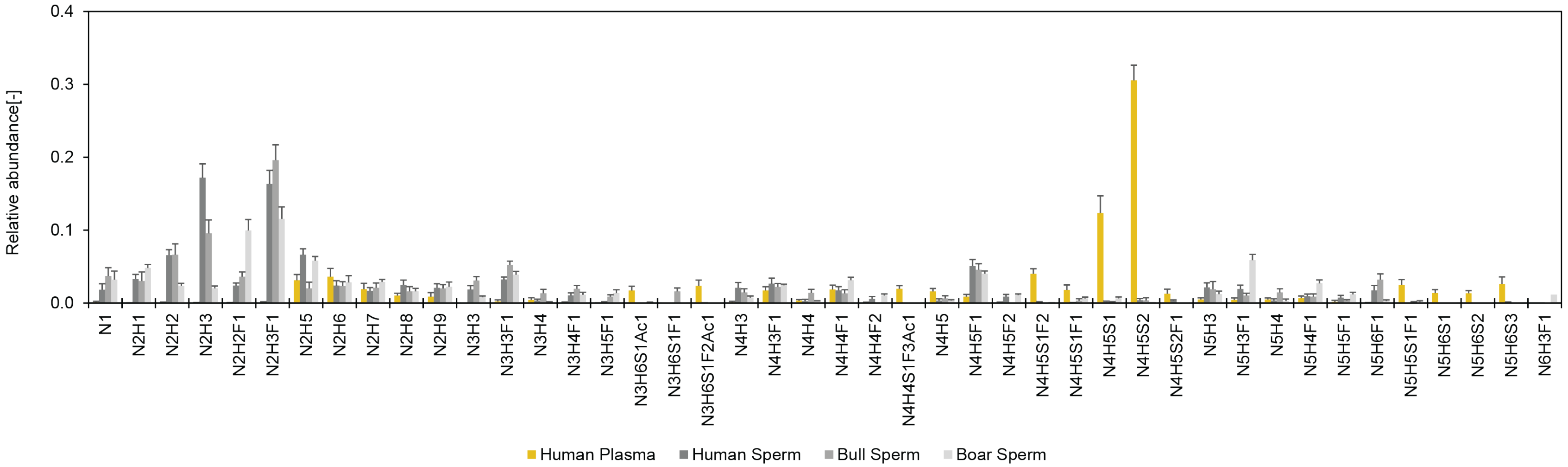

Supplementary Figure S8. Overview of *N*-glycan compositions observed in human, bull, and boar sperm cells, and for comparison in human plasma. The relative abundance of these glycan compositions was quantified on the number of detections identified using Byonic. The mean and standard deviation of the number were calculated using nine independent injections. Glycan composition was only included in the comparison when the relative abundance was higher than 0.01 for any species of that glycan composition. The bars show the mean ± standard deviation of the technical replicates of nine injections with different MS methods. N: *N*-acetylhexosamine; H: hexosamine; F: fucose; S: *N*-acetylneuraminic acid; Ac: acetylation.

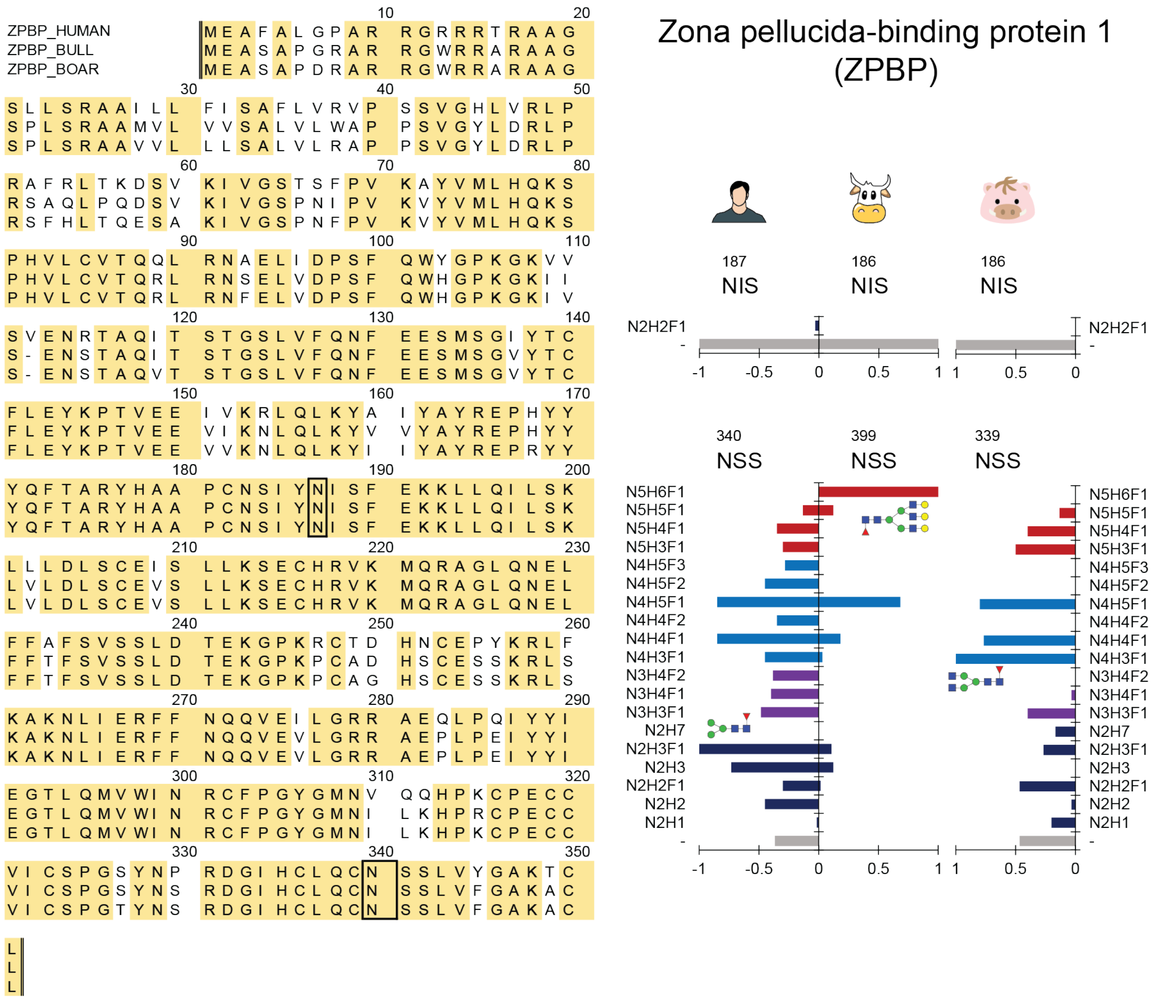

Supplementary Figure S9. Sequence alignment and site-specific *N*-glycan profile observed on zona pellucida-binding protein 1 (ZPBP1). ZPBP1 is highly abundant in sperm of human, bull and boar origin. The sequences of ZPBP1 are 86% identical across the three species (calculated by averaging the percent identity matrix) and two of the *N*-glycosylation sites (Asn187 and Asn340 in human) are also conserved. Moreover, the *N*-glycans decorating these two conserved sites are also similar, based on the glycoproteomics data generated here. In this analysis glycans were only included when at least five glycopeptide PSMs were detected at a glycosylation site. The protein sequences were aligned in UniProt with zero iterations and the same amino acids at the same sites of human, bull, and boar sperm cell proteins are highlighted in yellow. Observed *N*-glycosites are annotated in black boxes. Principally, proteins are listed by their gene name. For those proteins without gene name, UniProt entry name or identifiers were used instead.

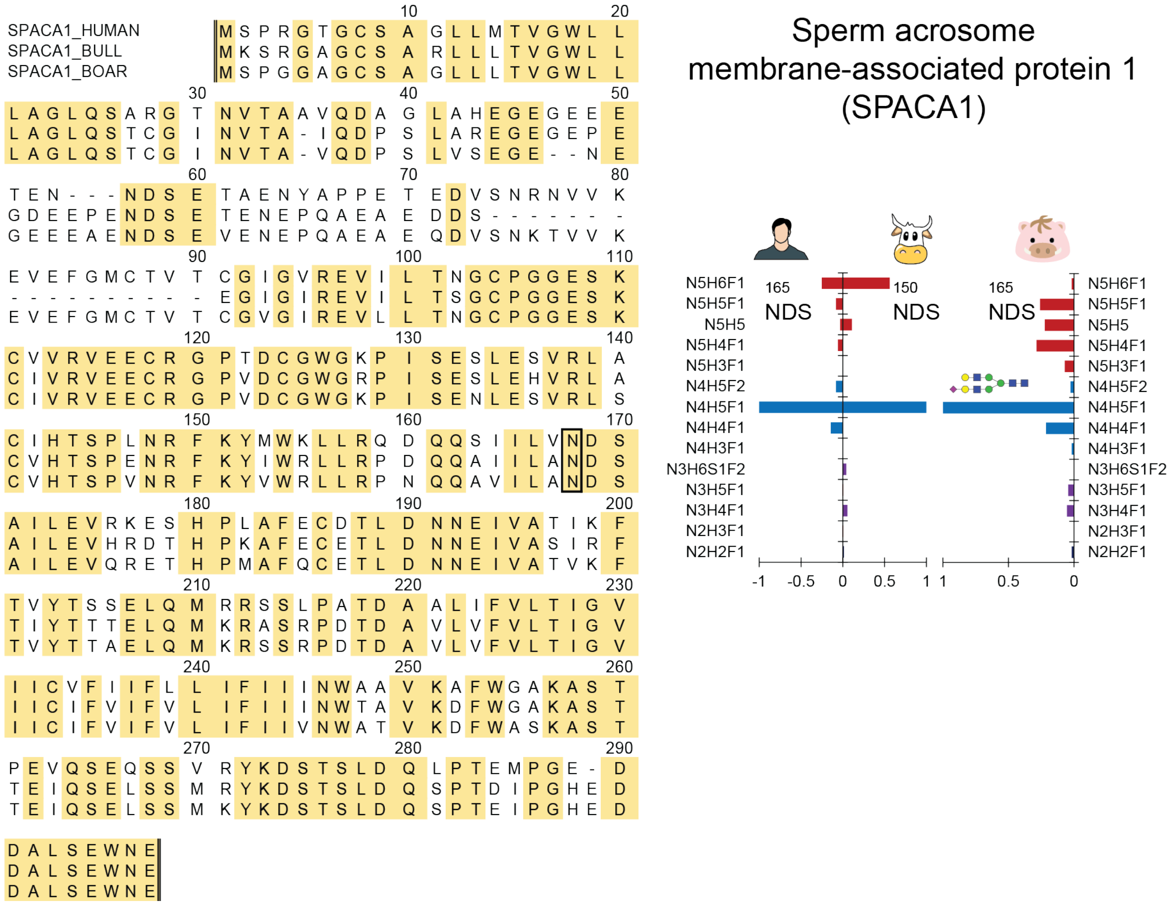

Supplementary Figure S10. Sequence alignment and site-specific *N*-glycan profile of sperm acrosome membrane-associated protein 1 (SACA1). SACA1 is highly abundant in sperm of human, bull and boar origin. The sequences of SACA1 are 78% identical across the three species (calculated by averaging the percent identity matrix) and the *N*-glycosylation site (Asn165 in human) is also conserved. Moreover, the *N*-glycans decorating this conserved site are also similar, based on the glycoproteomics data generated here. In this analysis glycans were only included when at least five glycopeptide PSMs were detected at a glycosylation site. The protein sequences were aligned in UniProt with zero iterations and the same amino acids at the same sites of human, bull, and boar sperm cell proteins are highlighted in yellow. Observed *N*-glycosites are annotated in black boxes. Principally, proteins are listed by their gene name. For those proteins without gene name, UniProt entry name or identifiers were used instead.

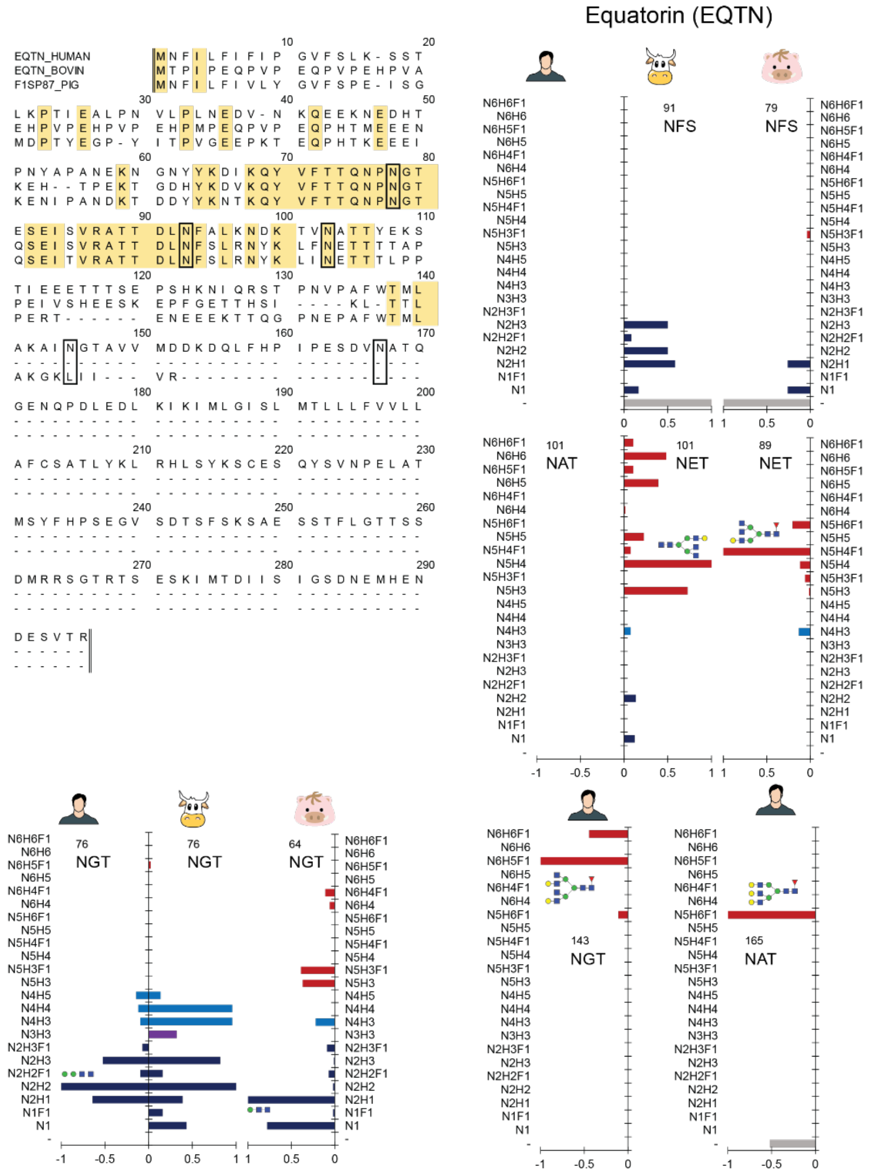

Supplementary Figure S11. Sequence alignment and site-specific *N*-glycan profile on equatorin (EQTN). The sequences of equatorin are 48% identical (calculated by averaging the percent identity matrix) and two out of five *N*-glycosylation sites (Asn76 and Asn101 in human) are also conserved. Moreover, the *N*-glycans decorating these two conserved sites are also similar, based on the glycoproteomics data generated here. Asn101 (in human) does not reside in a *N*-glycosylation motif (NXS/NXT, Xxx ≠ Pro) and human equatorin has two extra *N*-glycosylation sites at Asn143 and Asn165. In this analysis glycans were only included when at least five glycopeptide PSMs were detected at a glycosylation site. The protein sequences were aligned in UniProt with zero iterations and the same amino acids at the same sites of human, bull, and boar sperm cell proteins are highlighted in yellow. Observed *N*-glycosites are annotated in black boxes. Principally, proteins are listed by their gene name. For those proteins without gene name, UniProt entry name or identifiers were used instead.

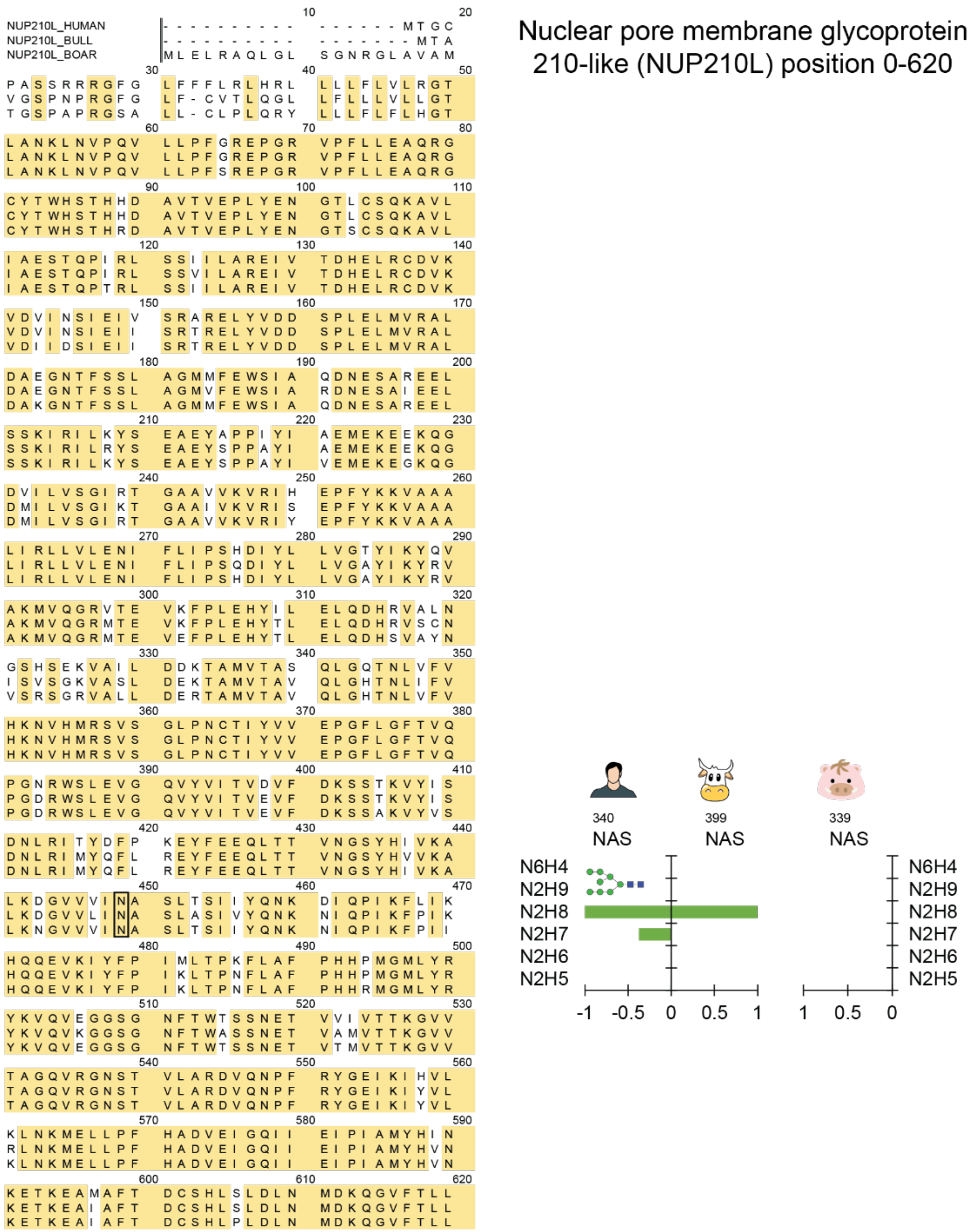

Supplementary Figure S12. Sequence alignment and site-specific *N*-glycan profile detected on nuclear pore membrane glycoprotein 210-like (P210L) position 0-620. The sequences of P210L are 90% identical across the three species (calculated by averaging the percent identity matrix) and the *N*-glycosylation sites (Asn340 in human) is also conserved. Moreover, the *N*-glycans decorating these two conserved sites are also similar, based on the glycoproteomics data generated here. In this analysis glycans were only included when at least five glycopeptide PSMs were detected at a glycosylation site. The protein sequences were aligned in UniProt with zero iterations and the same amino acids at the same sites of human, bull, and boar sperm cell proteins are highlighted in yellow. Observed *N*-glycosites are annotated in black boxes. Principally, proteins are listed by their gene name. For those proteins without gene name, UniProt entry name or identifiers were used instead.

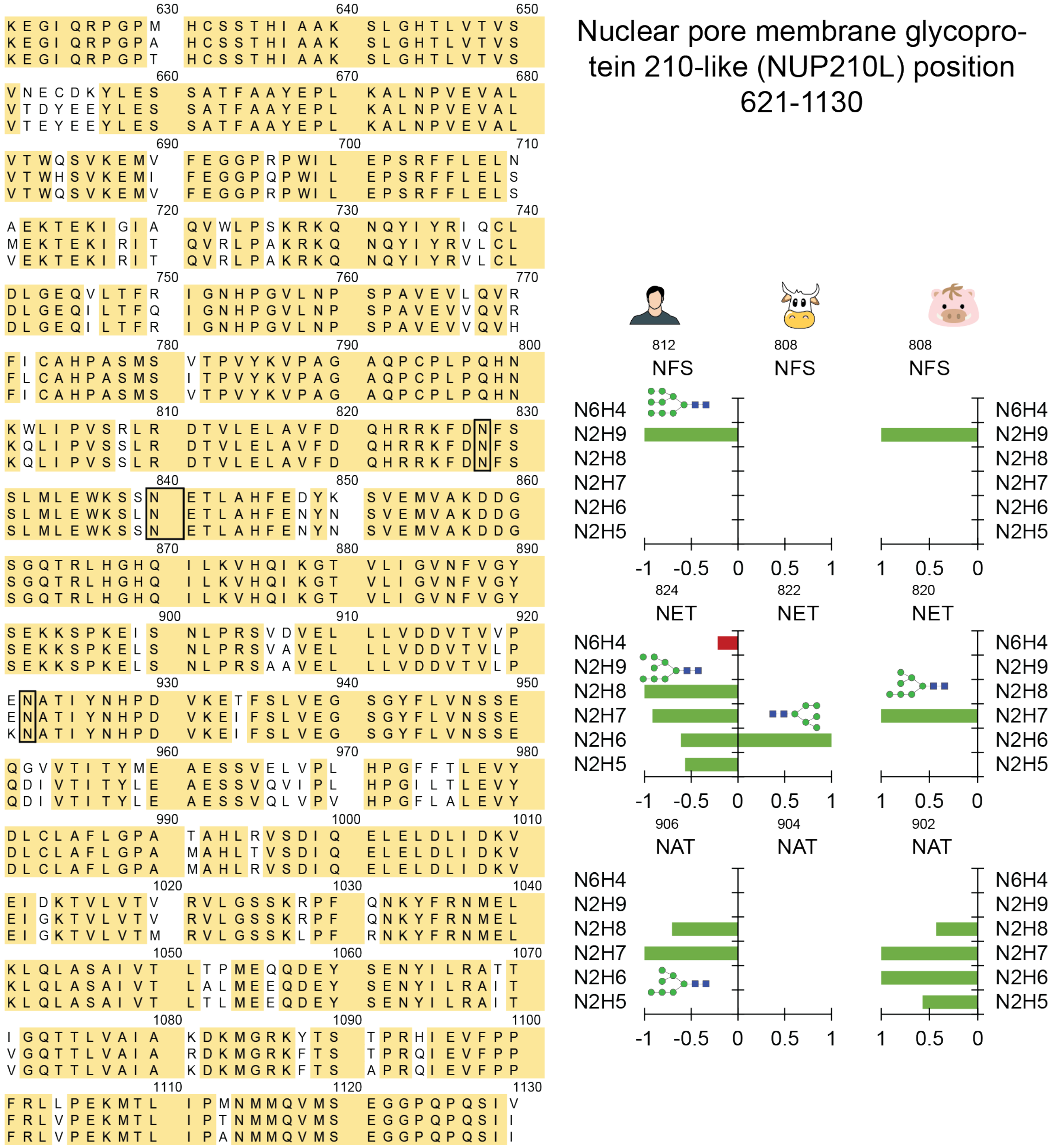

Supplementary Figure S13. Sequence alignment and site-specific *N*-glycan profile of nuclear pore membrane glycoprotein 210-like (P210L) position 621-1130. The sequences of P210L are 90% identical across the three species (calculated by averaging the percent identity matrix) and three of the *N*-glycosylation sites (Asn812, Asn824, and Asn906 in human) are also conserved. Moreover, the *N*-glycans decorating these two conserved sites are also similar, based on the glycoproteomics data generated here. In this analysis glycans were only included when at least five glycopeptide PSMs were detected at a glycosylation site. The protein sequences were aligned in UniProt with zero iterations and the same amino acids at the same sites of human, bull, and boar sperm cell proteins are highlighted in yellow. Observed *N*-glycosites are annotated in black boxes. Principally, proteins are listed by their gene name. For those proteins without gene name, UniProt entry name or identifiers were used instead.

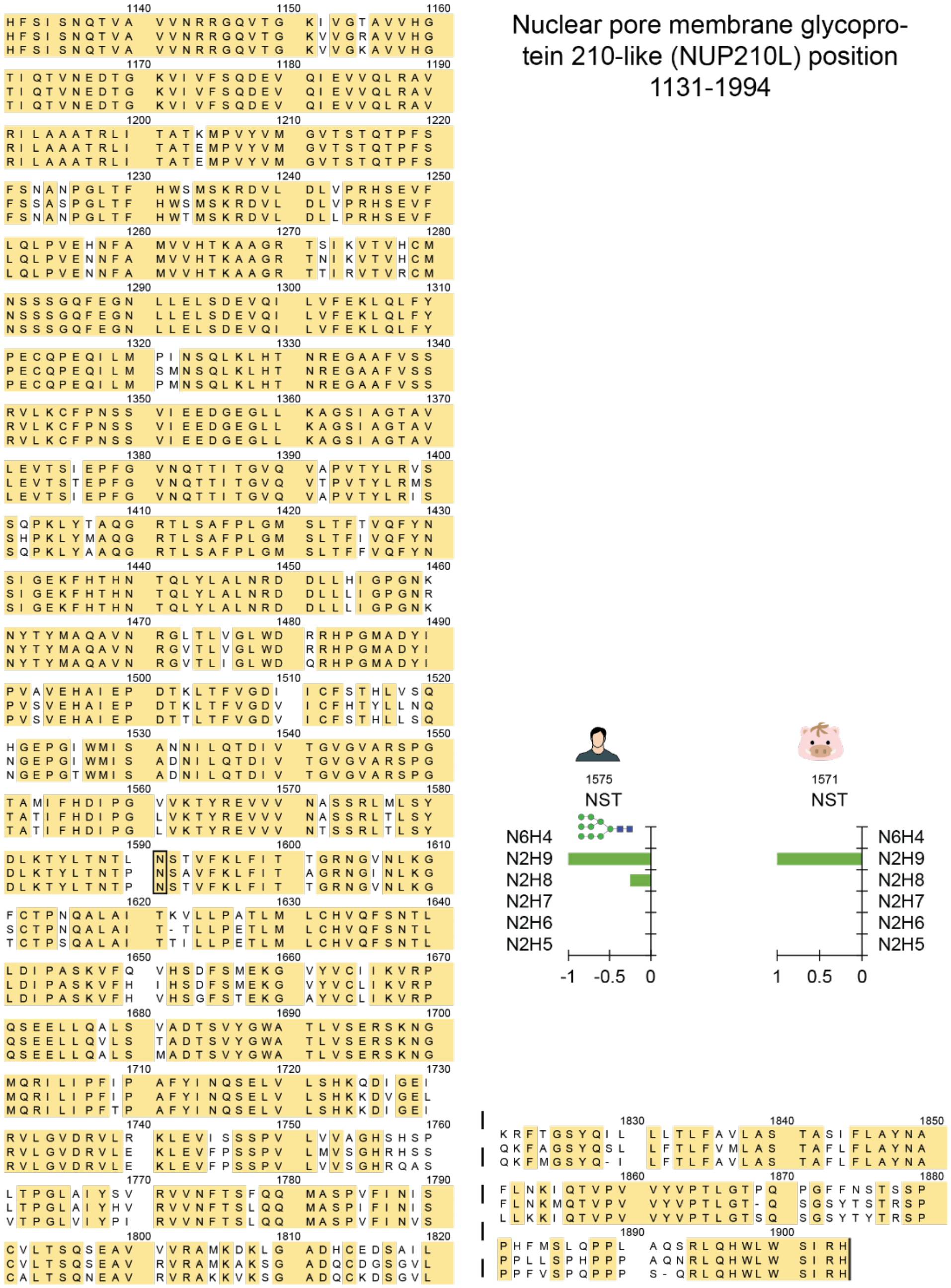

Supplementary Figure S14. Sequence alignment and site-specific *N*-glycan profile of nuclear pore membrane glycoprotein 210-like (P210L) position 1131-1994. The sequences of P210L are 90% identical across the three species (calculated by averaging the percent identity matrix) and the *N*-glycosylation sites (Asn1575 in human) is also conserved between human and boar. Moreover, the *N*-glycans decorating these two conserved sites are also similar, based on the glycoproteomics data generated here. In this analysis glycans were only included when at least five glycopeptide PSMs were detected at a glycosylation site. The protein sequences were aligned in UniProt with zero iterations and the same amino acids at the same sites of human, bull, and boar sperm cell proteins are highlighted in yellow. Observed *N*-glycosites are annotated in black boxes. Principally, proteins are listed by their gene name. For those proteins without gene name, UniProt entry name or identifiers were used instead.

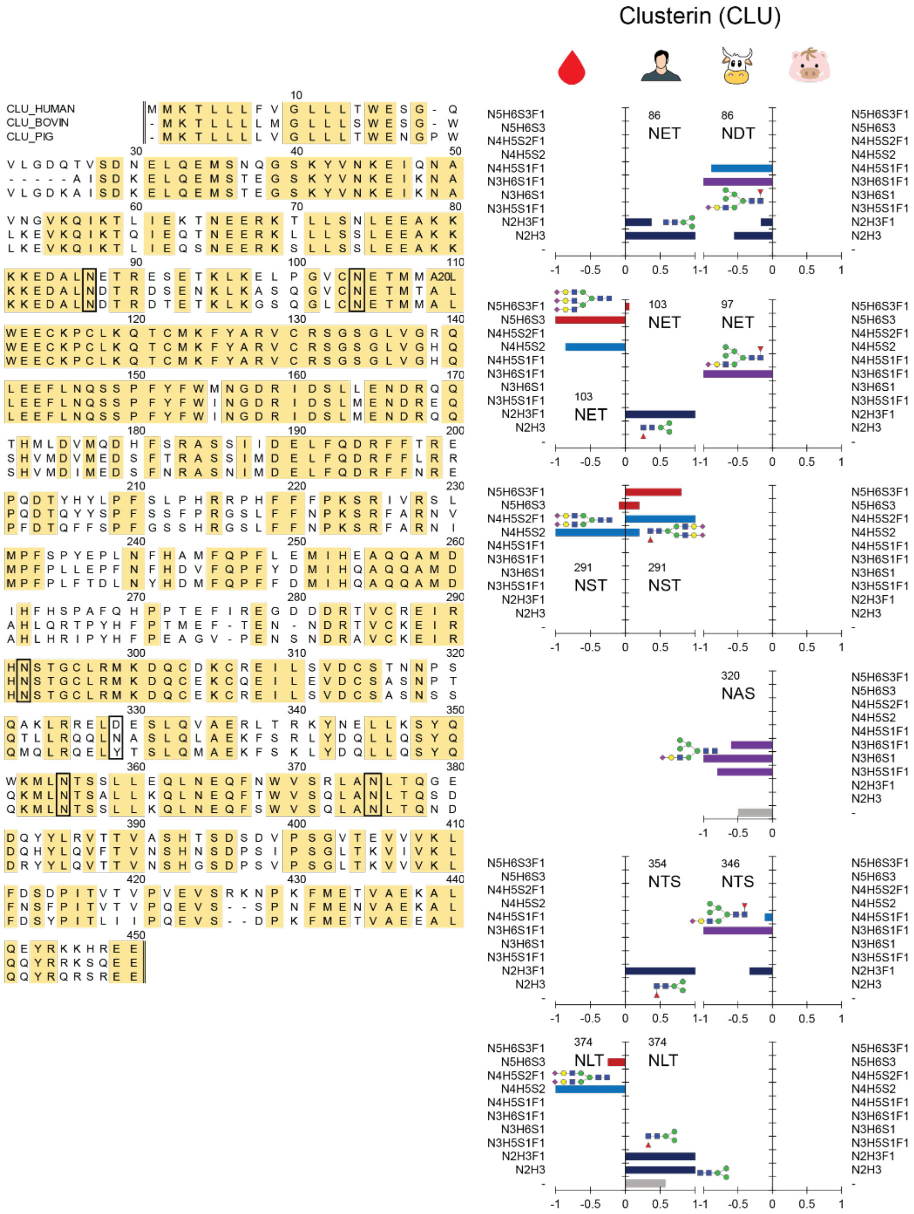

Supplementary Figure S15. Sequence alignment and site-specific *N*-glycan profiles of clusterin (CLUS). The sequences of clusterin are 76% identical across the three species (calculated by averaging the percent identity matrix). Five out of six *N*-glycosylation sites (Asn86, Asn103, Asn291, Asn354, and Asn374 in human) are also conserved. Bull clusterin has one more *N*-glycosylation site at Asn320. Moreover, the *N*-glycans decorating these five conserved sites are similar in terms of fucosylation among sperm cells while human plasma clusterin *N*-glycans are not fucosylated. In this analysis glycans were only included when at least five glycopeptide PSMs were detected at a glycosylation site. The protein sequences were aligned in UniProt with zero iterations and the same amino acids at the same sites of human, bull, and boar sperm cell proteins are highlighted in yellow. Observed *N*-glycosites are annotated in black boxes. Principally, proteins are listed by their gene name. For those proteins without gene name, UniProt entry name or identifiers were used instead.
